# Developing practical recommendations for drug-disease interactions in patients with hypertension

**DOI:** 10.3389/fphar.2024.1360146

**Published:** 2024-04-17

**Authors:** Kübra Özokcu, Maaike M. E. Diesveld, Suzan G. H. Gipmans, Laura E. J. Peeters, Bert-Jan van den Born, Sander D. Borgsteede

**Affiliations:** ^1^ Department of Hospital Pharmacy, Meander Medisch Centrum, Amersfoort, Netherlands; ^2^ Department of Hospital Pharmacy, Ziekenhuis Rivierenland, Tiel, Netherlands; ^3^ Department of Clinical Decision Support, Health Base Foundation, Houten, Netherlands; ^4^ Medicines Information Centre, Royal Dutch Pharmacists Association (KNMP), The Hague, Netherlands; ^5^ Department of Hospital Pharmacy, Maasstad Hospital, Rotterdam, Netherlands; ^6^ Departments of Internal Medicine and Public Health Amsterdam Cardiovascular Sciences Amsterdam UMC, Amsterdam, Netherlands

**Keywords:** hypertension, drug-disease interaction, clinical decision support, adverse drug reaction, blood pressure, product information, drug information

## Abstract

**Background:**

Hypertension, a significant risk factor for cardiovascular diseases, demands proactive management as cardiovascular diseases remain the leading cause of death worldwide. Reducing systolic and diastolic blood pressure levels below recommended reference values of <140/90 mmHg can lead to a significant reduction of the risk of CVD and all-cause mortality. However, treatment of hypertension can be difficult and the presence of comorbidities could further complicate this treatment. Drugs used to manage these comorbidities may inadvertently have an impact on blood pressure, resulting in a phenomenon known as drug-disease interaction. This study aims to assess the safety of medication that can affect blood pressure in patients with hypertension and provide practical recommendations for healthcare professionals.

**Methods:**

For the development of recommendations for the drug-disease interaction (DDSI) hypertension, a six-step plan that combined literature selection and multidisciplinary expert opinion was used. The process involved (1) defining the scope of the DDSI and selecting relevant drugs, (2) collecting evidence, (3) data-extraction, (4) reaching of expert consensus, (5) publication and implementation of the recommendations in healthcare systems and (6) updating the information.

**Results:**

An increase of 10 mmHg in systolic blood pressure and 5 mmHg in diastolic blood pressure was defined as clinically relevant. Corticosteroids, danazol, and yohimbine caused a clinically relevant DDSI with hypertension. Several other drugs with warnings for hypertension in the official product information were assessed to have no clinically relevant DDSI due to minor influence or lack of data on blood pressure. Drugs with evidence for a relevant change in blood pressure which are prescribed under close monitoring of blood pressure according to clinical guidelines, were deemed to be not clinically relevant for signalling.

**Conclusion:**

This study provides specific recommendations that can be implemented directly in clinical practice, for example, in clinical decision support systems, potentially resulting in safer drug use in patients with hypertension and better healthcare by reducing alert fatigue. Future research should focus on evaluating the effectiveness of implementation strategies and their impact on reducing unsafe use of medication in patients with hypertension.

## 1 Background

Cardiovascular diseases are the leading cause of deaths worldwide. In 2022, 22.9% of total deaths in the Netherlands could be attributed to cardiovascular diseases (Volksgezondheid en Zorg info, 2022). Hypertension is a key risk factor for cardiovascular diseases (Rapsomaniki et al., 2014; Flint et al., 2019; Centers for Disease Control and Prevention, National Center for Health Statistics, 2022; Volksgezondheid en Zorg info, 2022). According to the World Health Organization, hypertension affects 1.28 billion adults between 30 and 79 years worldwide (Mancia et al., 2023). From 1990 to 2019, the global prevalence of hypertension doubled among individuals aged 30–79 years leading to a prevalence of 34% in men and 32% in women in 2019. For children, the prevalence was estimated at 4.0% with an increasing prevalence in the last decades (Song et al., 2019). In the Netherlands, approximately 15% of the population reports high blood pressure in 2022 (Volksgezondheid en Zorg info, 2022).

Normally, systolic blood pressure (SBP) and diastolic blood pressure (DBP) are respectively below 120 and 80 mmHg in adults (Whelton et al., 2022; Mancia et al., 2023). Hypertension is defined as SBP values ≥ 140 mmHg and/or DBP ≥90 mmHg (Mancia et al., 2023). Uncontrolled blood pressure, defined by SBP of ≥130 mmHg or a DBP of ≥80 mmHg, requires proactive interventions (Whelton et al., 2022; Mancia et al., 2023). Lifestyle changes, complemented by drug therapy, are needed in managing this critical health concern in hypertensive patients. Studies have shown that reducing SBP and DBP to levels below recommended reference values of <140/90 mmHg can lead to a significant reduction of the risk of CVD and all-cause mortality (Ettehad et al., 2016; Bundy et al., 2017). For this reason, it is important to control hypertension to reduce the risk of cardiovascular death.

However, treatment of hypertension can be difficult as many factors such as stress, diet and non-adherence to antihypertensive drug treatment can influence blood pressure (Samadian et al., 2016). Moreover, most patients with hypertension have comorbidities, such as other cardiovascular diseases, diabetes or COPD. The drugs used to treat these other comorbidities - not related to blood pressure control - may have an effect on blood pressure. When pharmacotherapy used to treat a disease causes worsening of another disease, in this context hypertension, it is called a drug-disease interaction (DDSI) (van Tongeren et al., 2020).

DDSIs are often stated as a warning in the Summary of Product Characteristics (SmPC). However, these statements may not always be practical, since they are often poorly substantiated or not applicable in practice. For some comorbidities and conditions, such as renal impairment and cirrhosis, other sources can be consulted, but there are few guidelines for DDSIs available (van Tongeren et al., 2020). In the Netherlands, a best practice has been developed on how to systematically evaluate DDSIs and implement recommendations in clinical practice (van Tongeren et al., 2020; Diesveld et al., 2021). This best practice defines the patient population at risk, and evaluates specific drugs with respect to the clinical relevance of its influence on blood pressure.

According to the warnings in the SmPCs, healthcare professionals might want to avoid prescribing drugs with a DDSI to patients with hypertension, given the negative effects with respect to blood pressure. It can be difficult to recognize which drugs should be avoided in patients with hypertension. Official product information, handbooks, professional guidelines and original manuscripts may describe a change in blood pressure as an adverse reaction and consequently lists these drugs as a contra-indication or warn that the drug should be avoided in patients with hypertension. However, a shortcoming is that hypertension caused by drugs is often not further defined, neither is the extent of blood pressure change. Moreover, it is not always clear if adverse events and warnings shown in product information and other literature are clinically relevant. For physicians and pharmacists, this can be problematic because it is not clear what kind of action is needed to optimize medication safety for drugs prescribed to patients with hypertension. The aim of this study is to assess the safety of medication that can affect blood pressure in patients with hypertension and provide practical recommendations for healthcare professionals.

## 2 Materials and methods

**FIGURE 1:**
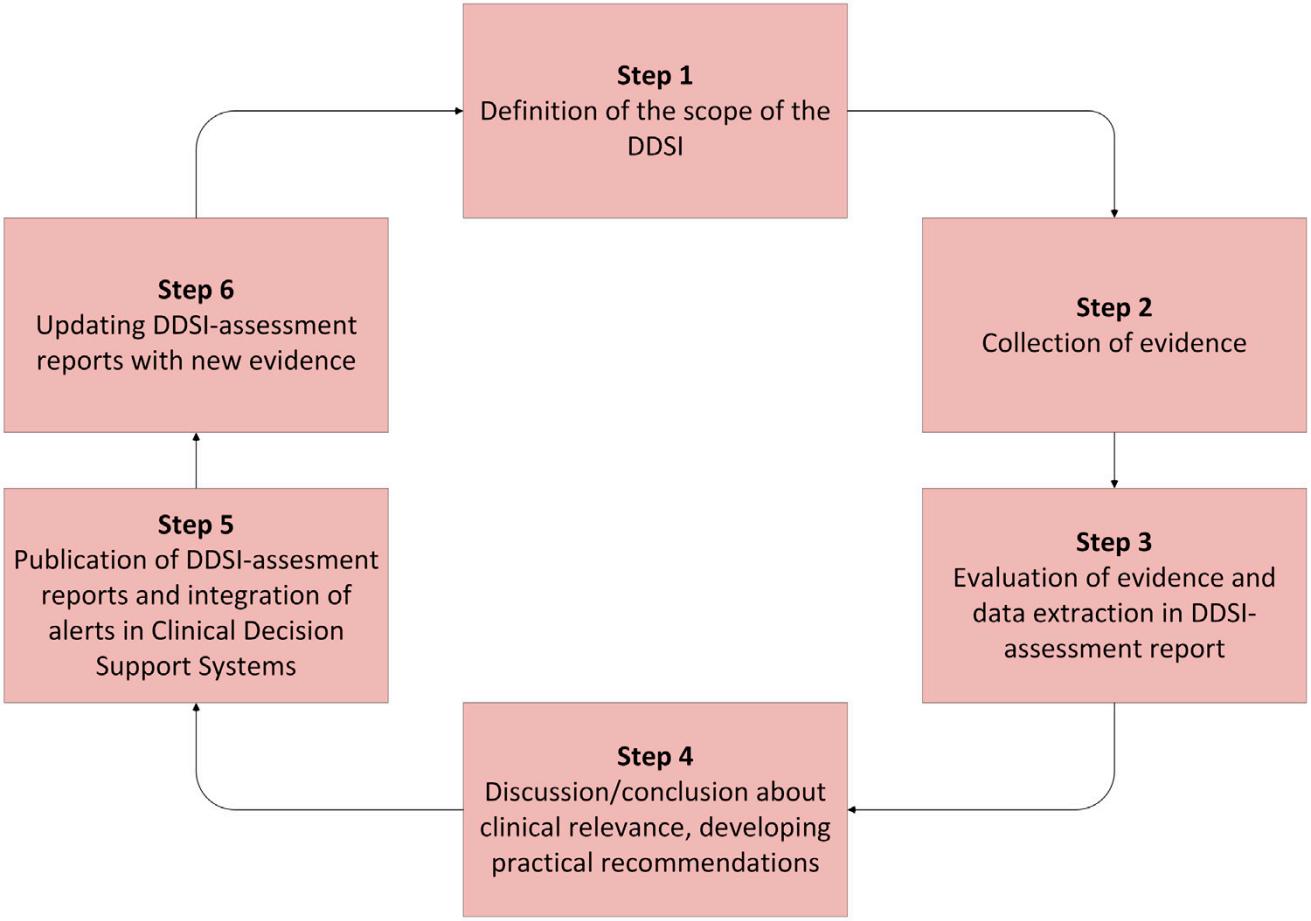
Flowchart of the six-step plan for evaluation of drug-disease interactions (DDSIs).

For the development of recommendations for the DDSIs of hypertension, we used a previously developed six-step plan and made it specific for hypertension (Figure 1). (van Tongeren et al., 2020) This method combines literature and expert opinion to develop new practical recommendations. Our expert panel consisted of a fixed multidisciplinary panel of nine members: four representatives of specialists responsible for prescribing: two internist-clinical pharmacologist and two general practitioners, five pharmacists responsible for dispensing: two hospital pharmacists and three community pharmacists, and in addition to the fixed panel three experts in the field of hypertension research and treatment: two vascular internists and one hospital pharmacist.

The six steps of the development of practical DDSI-recommendations were as described below:

### 2.1 Scope of the DDSI

The scope of the DDSI described the minimal change (rise and fall) in SBP and DBP that was regarded as clinically relevant. Subsequently, the drugs to be evaluated were selected. All drugs that mentioned hypertension in the Summary of Product Characteristics (rubric 4.3 “Contra-indications” and 4.4 “Warnings and precautions for use”) were selected.

### 2.2 Collection of evidence

Several sources were consulted for data with respect to the drugs identified in step 1. The European Society of Hypertension Guidelines (2023) and the harmonization (2022) of the American College of Cardiology/American Heart Association and 2018 European Society of Cardiology/European Society of Hypertension were consulted as clinical practice guidelines for management of high blood pressure (Whelton et al., 2022; Mancia et al., 2023). For all drugs, PubMed and EMBASE were searched for literature concerning these drugs with potential risks for patients with hypertension (see Table 1 for search strategy). Studies were eligible if they fulfilled the criteria for the PICO (Patient, Intervention, Comparison and Outcomes) model or described outcomes in the general population with respect to the influence of the drug on blood pressure. The key-question for the PICO was: does drug X affect blood pressure in patients with hypertension in comparison to placebo or does drug X increase the risk of side effects in patients with hypertension compared to patients without hypertension? The quality of the studies that were compliant with the PICO was evaluated with criteria for drug-drug interactions adjusted for DDSIs: grade 4 for highest level of evidence, grade 1 for lowest (van Roon et al., 2005). Publications with the highest level of evidence were included, as were clinically relevant studies with lower levels of evidence that described the population at risk. Based on previously available evidence in clinical decision support systems (CDSS), studies published in a timeframe of 2011–2023 were included. Studies were excluded if they did not involve humans.

**TABLE 1:** General search strategy, specified for each drug.


Pubmed	("Drug X"[Mesh] OR "Drug Y"[Mesh] OR "Drug Z"[Mesh]) AND ("Hypertension"[Mesh] OR "Blood Pressure"[Mesh])
Embase	('hypertension'/mj OR 'blood pressure'/mj) AND ('drug X'/mj OR 'drug Y'/mj OR 'drug Z) AND (‘placebo’/mj)

Moreover, the relevant rubrics in the European SmPCs and American FDA Prescribing Information describing contra-indications, warnings for special precaution, side effects and pharmacology were searched for information about the DDSI. Finally, national guidelines and handbooks describing hypertension were searched for information about the DDSI.

### 2.3 Data extraction

Data from the selected sources were extracted and presented in the DDSI assessment report. Essential information about the study type, number of patients with and without hypertension, drug dose, results and conclusions were summarized. Based on all the information collected, the assessment report included a preliminary conclusion on the clinical relevance of the DDSI and the proposed action in case of the DDSI.

### 2.4 Expert conclusion

The expert panel determined the scope of the DDSI, discussed the clinical relevance of the DDSIs, and made conclusions by consensus about the practical recommendations. If drugs met the criteria for clinical relevance, the expert panel decided to signal these DDSIs in CDSS according to clinically relevant literature and their expert opinion. The following reasons were distinguished for not signalling the DDSIs:

(1) Evidence that the specific drug had only a minor influence on blood pressure;
(2) No evidence (data) for an effect of the drug on blood pressure;
(3) Short term use with active monitoring of blood pressure;
(4) Drug is prescribed under close monitoring of blood pressure according to clinical guidelines.

The assessment reports were supplemented with the expert opinions and conclusions. The results of the assessment reports including the motivation of the expert panel for the reasons above and the underlying information in the SmPC and FDA Prescribing Information are presented in Tables 2–4. The references of the tables are included in Supplementary Material.

**TABLE 2:** Drugs evaluated as having a clinically relevant drug-disease interaction with hypertension, the underlying information in the SmPC and FDA product information, and the action recommended for healthcare professionals.

Therapeutic group	Mechanism of the DDSI	SmPC information	Product information (FDA)	Blood pressure increase	DDSI action
Corticosteroids	Mineralocorticoids act on the distal renal tubules and promote reabsorption of water and salt.	Corticosteroids should be administered with caution and under close monitoring in case of hypertension.(77)	Average and large doses of hydrocortisone or cortisone could cause elevation of blood pressure, salt and water retention, and increased excretion of potassium.(78)	≥30.0 mmHg SBP and >10.0 mmHg DBP.(21–28)	Close monitoring of blood pressure, adjust antihypertensive treatment if needed.
	Other mechanisms of action are: a direct effect on smooth muscle tissue of blood vessels, activation of the central and peripheral nervous system, increased blood vessel sensitivity to catecholamines, redistribution of body fluids with increased plasma volume and increased cardiac output.(76)				
Danazol	Weak mineralocorticoid action, which causes salt and water retention, increased plasma volume and increased cardiac output.(79)	Danazol may cause some fluid retention, so it is recommended to use danazol with caution in patients with hypertension.(80)	Because danazol may cause some degree of fluid retention, conditions that might be influenced by this factor, such as hypertension require careful observation.(81)	21.0 mmHg SBP and 20.0 mmHg DBP. (29–33)	Close monitoring of blood pressure, adjust antihypertensive treatment if needed.
Yohimbine	Selectively inhibits the alpha-2 receptor, thereby stimulating sympathetic activity in the central nervous system. This could lead to an increase in blood pressure and heart rate.(31)	The use of yohimbine is not recommended in patients with hypertension or hypotension.(82)	-	10.0 mmHg SBP and DBP.(31–33)	Close monitoring of blood pressure.

The references of this table are cited in Supplementary Material.

**TABLE 3:** Drugs evaluated as having no clinically relevant drug-disease interaction with hypertension, the underlying information in the SmPC and FDA product information, and the motivation of the expert panel for this conclusion.

Therapeutic group/drugs	Mechanism of the DDSI	SmPC information	Product information (FDA)	BP change
Evidence that the specific drug had minor influence on blood pressure				
Amphetamines	Stimulation of the release of norepinephrine and dopamine, especially in the central nervous system, resulting in an increase in blood pressure and heart rate.(83)	Treatment could lead to slight increase in blood pressure (approx. 2–4 mmHg). Caution in patients whose medical conditions may present a risk of an increase in blood pressure.(84)	Central nervous system (CNS) stimulants cause an increase in blood pressure (about 2–4 mmHg). Monitor all patients for potential hypertension.(85)	Increase of SBP and DBP of 1.2–4.0 mmHg. (86–90)
Androgens	Several mechanisms: vasoconstriction, atherosclerosis, stimulation of the renin-angiotensin-aldosterone system (RAAS), upregulation of thromboxane A2 expression, norepinephrine synthesis, angiotensin II expression, endothelin-1 stimulation, release of neuropeptide Y and attenuation of adenosine.(91–95)	Testosterone could cause an increase in blood pressure. This drug should be used with caution in men with hypertension.(96)	After initiating therapy, periodically monitor for and treat new-onset hypertension or exacerbations of pre-existing hypertension.(97)	Increase of 0.9 mmHg SBP and 2.0 mmHg DBP. (98–106)
Antidepressants	Affection of vagal control of the heart, which has been seen to the greatest extent for TCAs. This could possibly lead to increased blood pressure.(107)	Adverse reaction: vascular disease; hypertension.(109)	Adverse reaction: cardiovascular; hypertension.(112,113)	Increase of 1.8 mmHg SBP and 1.6 mmHg DBP. (39–41)
*Tricyclic*				
*Antidepressants (TCAs*)				
*Specific Serotonin*	The underlying mechanism remains unclear. Several mechanisms: regulation of vascular tone by serotonin, inhibition of the synthesis of nitric oxide and reduction of sympathetic nervous system.(108)	Overdose: Hypertension is observed.(110)	Blood pressure should be measured prior to initiating treatment and periodically measured throughout treatment.(114)	Decrease of 13.0 mmHg SBP and 8.6 mmHg DBP. (34,36–38,107,115)
*Reuptake Inhibitors (SSRIs*)		Caution is advised in patients where underlying diseases may be exacerbated by increases in blood pressure. Blood pressure should be monitored.(111)		
Atomoxetine	Inhibition of the norepinephrine transporter theoretically resulting in increased norepinephrine plasma levels and increased blood pressure.(116)	Caution is advised in patients whose underlying medical condition may be worsened by increases in blood pressure and heart rate.(117)	Caution is advised in patients whose underlying medical conditions could be worsened by increases in blood pressure or heart rate. Pulse and blood pressure should be measured at baseline, at dose increases and periodically.(118)	Increase of 2.9 mmHg SBP and 2.1 mmHg DBP. (87,88,116)
Hormonal contraceptives	Stimulation of renin synthesis by estrogens causing water and salt retention and peripheral vasoconstriction.(119)	No association has been demonstrated between the use of hormonal contraceptives and clinical hypertension.(120)	Closely monitoring is advised and if significant elevation of blood pressure occurs, oral contraceptives should be discontinued.(121)	Increase of 5.8 mmHg SBP and 3.6 mmHg DBP. (122–124)
Metyrapone	Inhibition of steroid 11β-hydroxylase, resulting in decreased cortisol and corticosterone production. This could lead to a blood pressure lowering effect.(79)	Prolonged treatment with Metopirone may cause hypertension due to excessive secretion of desoxycorticosterone.(125)	Treatment and Management of Overdosage: For a few days blood pressure and fluid and electrolyte balance should be monitored.(126)	The extent of blood pressure decrease is unknown. (127)
Mirabegron	Selective beta-3 agonist, which loses its selectivity at higher doses (>100 mg) leading to activation of beta-1 adrenoreceptors in the cardiovascular system.(128)	Blood pressure should be measured at baseline and periodically during treatment with mirabegron, especially in hypertensive patients.(129)	Periodic blood pressure determinations are recommended, especially in hypertensive patients.(130)	Increase of 9.3 mmHg (SBP). (128,131–134)
NSAIDs (incl. selective COX-2-inhibitors)	Several mechanisms: sodium retention, decreased prostaglandin-induced renal vasodilation, vasoconstriction.(79,135)	Caution is advised in patients with a history of hypertension failure prior to treatment. Fluid retention, hypertension and edema have been reported in association with NSAID therapy.(136)	NSAIDs could lead to new onset of hypertension or worsening of pre-existing hypertension.(137)	Increase of 6.0 mmHg. (138–140)
Thiazolidinediones	Occurrence of oedema which theoretically could result in an increase in blood pressure.(141)	-	-	Increase of 4.7 mmHg SBP and 1.7 mmHg DBP. (142)
Thyromimetics	The underlying mechanism remains unknown. Genetic mutations in the hypothalamic-pituitary-thyroid system may have an effect on blood pressure. Thyroid dysfunction can cause atherosclerotic changes can cause elevated blood pressure.(143)	Hypertension should be excluded or treated before starting treatment with thyroid hormones.(144)	Adverse reactions: increased blood pressure.(145)	Reduction of 7.7 mmHg SBP and 4.5 mmHg DBP. (146)
*No evidence (data*) *for an effect of the drug on blood pressure*				
Antithrombotic drugs	Antithrombotics are associated with a risk of (intracerebral) bleeding, which may be increased by hypertension.(147) No effect on blood pressure has been described.	The risk of bleeding may be increased in patients with uncontrolled severe arterial hypertension and/or receiving concomitant therapy affecting hemostasis.(148)	-	*No evidence (data) for an effect of the drug on blood pressure.*
Disulfiram	Unclear, disulfiram could decrease norepinephrine levels.(149)	-	-	Increase of 48.9 mmHg SBP and 24.0 mmHg DBP. (150–152)
Methylergometrine	Partial agonist and antagonist of serotonergic, dopaminergic and adrenergic receptors. Weak effect on peripheral vessels.(153)	Close monitoring of blood pressure should be considered in case of mild or moderate hypertension (severe hypertension is contraindicated).(153)	Methylergometrine should be given slowly with careful monitoring of blood pressure, because of the possibility of inducing sudden hypertensive and cerebrovascular accidents.(154)	*No evidence (data) for an effect of the drug on blood pressure.* (155)
*Short term use of the drug of interest to cause a change in blood pressure*				
Imidazoline derivatives (nafazoline, oxymetazoline, xylometazoline)	Stimulation of α1-adrenergic receptors, resulting in vasoconstriction of blood vessels.(79)	Caution is advised in patients with hypertension.(156)	Ask a doctor before use if you have high blood pressure.(157)	The extent of blood pressure increase is unknown.
Prolactin inhibitors (bromocriptine, cabergoline)	Direct precursor to norepinephrine, which at high doses also stimulates the alpha receptors, causing vasoconstriction.	Periodic monitoring of blood pressure is recommended in patients taking bromocriptine.(158)	Periodic monitoring of the blood pressure, particularly during the first weeks of therapy is prudent.(159)	Increase of 34.0 mmHg. (42–46)
Sympathomimetics alpha + beta (dopamine, ephedrine, epinephrine, pseudo-ephedrine)	Stimulation of the adrenergic receptors at the blood vessels and a release of norepinephrine, which stimulates α1- and β1-receptor, resulting in vasoconstriction.(160)	Caution is advised in patients with hypertension.(161,162)	Careful monitoring is advised for patients with hypertension.(165,166)	Increase of 10.0 mmHg (ephedrine nasal, epinephrine epidural, pseudo-ephedrine nasal). (168,169)
		During treatment, blood pressure should be monitored.(163,164)	When used to prevent hypotension, ephedrine has been associated with an increased incidence of hypertension.(167)	
Sympathomimetics beta (dobutamine, formoterol, isoprenaline, salbutamol)	Increase in blood pressure via the beta-1 effect. Drugs with a predominantly beta-2 effect, on the other hand, cause vasodilation	Caution is advised in patients with hypertension.(170,171)	Caution is advised in patients with hypertension.(174)	Increase in blood pressure ≥50 mmHg.(172)
		Increase in blood pressure ≥50 mmHg.(172)	Patients with pre-existing hypertension appear to face an increased risk of developing an exaggerated pressor response.(175)	
		Overdose: increased systolic pressure and decreased diastolic pressure may occur.(173)		
Triptans and ergotamine	Vasoconstriction via the 5-HT1B/1D receptor.(176)	Caution is advised in patients with mildly controlled hypertension, as transient increases in blood pressure and peripheral vascular resistance have been observed in a small number of patients.(177)	Caution is advised in patients with controlled hypertension as transient increases in blood pressure and peripheral vascular resistance have been observed in a small proportion of patients.(178)	Increase of 22.0 mmHg SBP and 13.0 mmHg DBP. (179–181)

BP, blood pressure; DBP, diastolic blood pressure; DDSI, drug-disease interaction; SBP, systolic blood pressure; SmPC, summary of product characteristics. The references of this table are cited in Supplementary Material.

**TABLE 4:** Drugs evaluated as having a drug-disease interaction with hypertension, but are deemed to be not clinically relevant because of close monitoring of blood pressure according to clinical guidelines and the underlying information in the SmPC and FDA product information.

Therapeutic group/drugs	Mechanism of the DDSI	SmPC information	Product information (FDA)	BP change (maximum)
Alemtuzumab	Rapid depletion of lymphocytes due to alemtuzumab, resulting in induction of cytokine release. As a consequence, systemic proinflammatory reaction may induce the increase of SBP.(182)	Adverse reaction: hypertension.(183)	Infusion reactions: hypertension, hypotension.(184)	Increase of 19.2 mmHg SBP and 6.2 mmHg DBP. (185)
Angiogenesis inhibitors (lenalidomide, pomalidomide, thalidomide)	The underlying mechanism is unknown.	Adverse reaction: hypotension, hypertension	Adverse reaction: hypotension, hypertension.	The extent of blood pressure decrease/increase is unknown.
		Caution is advised in patients with known risk factors for thromboembolism (hypertension).(186)	Caution is advised in patients with known risk factors for thromboembolism (hypertension).(187)	
Basiliximab	The underlying mechanism is unknown. Probably occurrence of oedema which theoretically could result in an increase in blood pressure.	Adverse reaction: hypertension.(188)	Adverse reaction: hypertension.(189)	The extent of blood pressure increase is unknown. (190)
Bortezomib, carfilzomib)	Decrease in nitric oxide availability and endothelial dysfunction causing hypertension.(5)	Adverse reaction: hypotension, hypertension.	Adverse reaction: hypotension, hypertension.	The extent of blood pressure decrease/increase is unknown.
		Treatment with bortezomib is associated with orthostatic hypotension.(191)	Caution is advised in patients using antihypertensives.(192)	
Calcineurin inhibitors	Increase in blood pressure by affecting physiological systems (↑ RAAS system and endothelin-1, ↑ sympathetic nerve system, ↑ nitric oxide system).(5,55)	Blood pressure should be monitored regularly during treatment with cyclosporin. If hypertension occurs, appropriate antihypertensive therapy should be instituted.(193)	Patients who may be at increased risk such as those with abnormal renal function, uncontrolled hypertension or malignancies, should not receive cyclosporine capsules.(194)	Increase of 15.0 mmHg. (49–55)
Erythropoietic growth factors	Unclear, may be caused by an increase in vascular resistance due to vasoconstrictive effects of endothelin-1 and a decrease in vasodilating nitric oxide.(195)	Blood pressure should be closely monitored and treated as necessary in all patients treated with epoetin alfa. Initiation or increase of antihypertensive therapy may be necessary.(196)	Appropriately control hypertension prior to initiation of and during treatment with epoetin-alfa. Reduce or withhold epoetin-alfa if blood pressure becomes difficult to control.(197)	Increase of >10 mmHg. (195,198–202)
Immunomodulants (abatacept, glatiramer, vedolizumab)	The underlying mechanism is unknown.	Adverse reaction: hypertension, hypotension.(203)	Adverse reaction: hypertension, hypotension.(204)	The extent of blood pressure increase/decrease is unknown.
Leflunomide, Teriflunomide	The underlying mechanism is unknown. Teriflunomide is an active metabolite of leflunomide.	Blood pressure should be monitored at the start of leflunomide treatment and periodically thereafter.(205)	Blood pressure should be checked before starting treatment with leflunomide and monitored periodically thereafter.(207)	Leflunomide: Increase of 40.0 mmHg SBP and 20.0 mmHg DBP.
		Elevation of blood pressure may occur during treatment with teriflunomide. Blood pressure elevation should be appropriately managed before and during treatment with teriflunomide.(206)	Teriflunomide may increase blood pressure. Measure blood pressure at treatment initiation and monitor blood pressure during treatment.(208)	Teriflunomide: the extent of blood pressure increase is unknown. (21,209–212)
Mono-Amine Oxidase (MAO) inhibitors (classic)	Prevention of the metabolism of tyramine, causing a rise in tyramine levels in blood and therefore a rise in blood pressure.(79)	Patients with moderately elevated or low blood pressure or those at increased risk of hypertensive reactions (e.g., hyperthyroidism) should only take tranylcypromine with regular blood pressure monitoring.(213)	Assess blood pressure before prescribing tranylcypromine tablets and closely monitor blood pressure in all patients taking tranylcypromine tablets.(214)	Increase of 42.0 mmHg. (56–58,61–63)
MTOR-inhibitors (everolimus, sirolimus, temsirolimus)	Decrease in VEGF bioavailability causing hypertension.(5)	Adverse reaction: hypertension.(215)	Adverse reaction: hypertension.(216)	Decrease of 13.0 mmHg. (217,218)
Phenethylamine derivatives (phenylephrine, midodrine)	Stimulation of α1-adrenergic receptors, resulting in vasoconstriction of blood vessels and an increase in blood pressure.(79)	Regular monitoring of blood pressure is necessary because of the risk of hypertension in the lying position, e.g., in the evening.(219)	Blood pressure should be monitored carefully when midodrine hydrochloride is used concomitantly with other agents that cause vasoconstriction.(220)	Increase of >15.0 mmHg for phenylephrine, >20.0 mmHg for midodrine. (221–229)
Phosphodiesterase inhibitors (milrinon, enoximon)	Selective inhibition of the enzyme phosphodiesterase 5 (PDE 5), which catalyzes the hydrolysis of cyclic guanosine monophosphate (cGMP), causing vasodilation.(230)	Caution is advised in patients who have low blood pressure prior to treatment, because milrinone may cause hypotension due to its vasodilatory action.(231)	During therapy with milrinone, blood pressure and heart rate should be monitored and the rate of infusion slowed or stopped in patients showing excessive decreases in blood pressure.(232)	The extent of blood pressure decrease is unknown. (233,234)
Prostaglandins	Elicitation of natriuresis, water diuresis, causing an increase in renal blood flow and a decrease in systemic blood pressure.(235)	-	Adverse reaction: causal relationship unknown; hypertension.(236)	Increase of 16.0 mmHg SBP and 19.0 mmHg DBP. (237,238)
Protein kinase inhibitors (binimetinib, brigatinib, erlotinib, gefitinib, lorlatinib)	Effect on the RAAS system and decreased nitric oxide availability due to effect on vascular endothelial growth factor (VEGF) pathway causing an increase in blood pressure.(239)	Blood pressure should be measured at baseline and during treatment, with management of high blood pressure by standard therapy as appropriate.(240)	Before taking binimetinib, tell your healthcare provider about all of your medical conditions, including if you have high blood pressure (hypertension).(241)	The extent of blood pressure increase is unknown. (242–246)
TNF-alpha inhibitors	The underlying mechanism remains unknown. Serum Tumor necrosis factor-alpha (TNF-a) level is elevated in hypertensive patients.(247) Theoretically the use of TNF-alpha inhibitors could lead to a decrease in blood pressure	Adverse reactions - Common: Hypertension.(248)	Serious cerebrovascular accidents, myocardial ischemia/infarction (some fatal), hypotension, hypertension, and arrhythmias have been reported during and within 24 h of initiation of infliximab infusion.(249)	Increase of 2.0 mmHg SBP and 1.0 mmHg DBP. (247,250–253)
VEGF-antagonists (aflibercept, axitinib, bevacizumab, cabozantinib, dasatinib, lenvatinib, nilotinib, nintedanib, pazopanib, ponatinib, ramucirumab, regorafenib, sorafenib, sunitinib, tivozanib, vandetanib)	Decreased production and release of nitric oxide and prostacyclin and increased vascular resistance and blood pressure.(254) Increase in endothelin-1 bioavailability, oxidative stress and dysfunction of endothelial microvascular rarefication.(5) Also an alteration in glomerular filtration, loss of capillary circulation and imbalance between vasodilators and vasoconstrictors.(255)	It is recommended to monitor blood pressure every 2 weeks or more often during treatment.(256)	Monitor blood pressure every 2 weeks of more often during treatment. (257)	The extent of blood pressure decrease is unknown. (258)

BP, blood pressure; DBP, diastolic blood pressure; DDSI, drug-disease interaction; SBP, systolic blood pressure; SmPC, summary of product characteristics. The references of this table are cited in Supplementary Material.

### 2.5 Publication and implementation

The conclusions and recommendations were published in the Dutch standards for Clinical Decision Support (Borgsteede et al., 2023; Royal Dutch Pharmacists Association and Koninklijke Nederlandse Maatschappij ter bevordering der Pharmacie, 2023) and were implemented nationwide in all Healthcare Information Systems in primary and secondary care. The assessment reports have been published (in Dutch) and can be consulted in the Dutch standards for Clinical Decision Support: The G-Standaard and Pharmabase. An example of a translated assessment report is included as Supplementary Material. For clinically relevant DDSIs, an alert is generated automatically in CDSS when the drug of interest is prescribed to a patient labelled with hypertension.

### 2.6 Updating

The last step is to keep the information up-to-date after implementation. New drugs will be evaluated once they have market authorization, and literature and risk information will be screened periodically for every single drug.

## 3 Results

### 3.1 Scope: hypertension as drug-disease interaction

The degree of changes in blood pressure is important for the DDSI hypertension. The meta-analysis by Whelton et al. reported that an increase of 20 mmHg for SBP and an increase of 10 mmHg for DBP was associated with an increased cardiovascular disease risk (Whelton et al., 2017). The expert panel defined an increase of >10 mmHg of SBP and >5 mmHg of DBP as clinically relevant, which could already increase the risk of cardiovascular diseases (Vishram et al., 2012; Whelton et al., 2017; Williams et al., 2018). For decreases in blood pressure, a reduction of at least 20 mmHg SBP and 10 mmHg DBP was defined as clinically relevant according to the European hypertension guideline with regard to orthostatic hypotension (Williams et al., 2018).

The databases were searched for the most recent evidence published until 2023. The drugs that were evaluated are listed in Table 2 (drugs with a clinically relevant DDSI), Table 3 (drugs with no clinically relevant DDSI) and Table 4 (drugs with close monitoring of blood pressure according to clinical guidelines). Table 2 also presents the conclusions and suggested recommendations on how to act when the DDSI occurs. Furthermore, motivations were given for not recommending an alert for a certain DDSI (Tables 3, 4). Below, the clinically relevant drugs for DDSI hypertension are described.

### 3.2 Clinically relevant drug-disease interactions

#### 3.2.1 Corticosteroids

Corticosteroids can cause or exacerbate hypertension, especially when used for more than 2 weeks and in high doses (daily dose of prednisone equivalent >7.5 mg) (Distler et al., 1979; Panoulas et al., 2008; Sazliyana et al., 2011; Fardet et al., 2015; Miyabe et al., 2016; Baker et al., 2018; Bloechliger et al., 2018; Rice et al., 2018).

The advice for all high dosed corticosteroids that are used for more than 2 weeks is to monitor blood pressure more often. If necessary, the antihypertensive therapy has to be adjusted according to the blood pressure.

#### 3.2.2 Danazol

Danazol has a weak mineralocorticoid activity, through which it can increase blood pressure via water and salt retention. A clinically relevant increase in blood pressure of approximately 20 mmHg has been observed after use of danazol in two case reports (Bretza et al., 1980; Pears and Sandercock, 1990). It is recommended to monitor blood pressure more often and to adjust the antihypertensive therapy if the blood pressure remains high during danazol treatment.

#### 3.2.3 Yohimbine

Yohimbine is an α2-adrenergic receptor antagonist, which stimulate sympathetic activity in the central nervous system and is generally used for impotence. Studies have shown that in patients with pre-existing hypertension, blood pressure increased more than in patients who did not have hypertension prior to yohimbine administration (Damase-Michel et al., 1993; Grossman et al., 1993; Musso et al., 1995). For this reason, yohimbine has to be replaced by a phosphodiesterase-5 inhibitor in patients with hypertension.

### 3.3 Monitoring of blood pressure

Monitoring of blood pressure may be part of clinical follow-up by the prescriber, yet self-monitoring is becoming more common and recommended by hypertension guidelines. With a validated blood pressure measurement device and instructions, it is possible to correctly monitor blood pressure at home (Whelton et al., 2017). In case of a clinically relevant DDSI, blood pressure should be monitored at least once a month after initiating therapy, subsequently every 3 months until blood pressure stabilizes. After stabilization monitoring can take place every 6 months.

### 3.4 Drug-disease interactions evaluated as not clinically relevant

Many sources, mainly the official product information, stated that certain drugs should be avoided or used with caution in patients with hypertension. Yet, after evaluation of the available evidence by the expert panel it was concluded that most drugs had no clinically relevant DDSI. These drugs without a clinically relevant effect on blood pressure are listed in Table 3. Below, a summary of recommendations of drugs without a clinically relevant DDSI for hypertension are combined and presented according to the reason.

#### 3.4.1 Evidence that the specific drug had minor influence on blood pressure

For a group of drugs, hypertension is described as a contra-indication in the SmPC. For some drugs (e.g., SSRI’s), no evidence was found in literature to support a clinically relevant DDSI for hypertension, nor was a plausible pharmacological mechanism described. For other drugs (e.g., amphetamines, hormonal contraceptives and thyromimetics), there was a plausible pharmacological mechanism, yet the magnitude of blood pressure change was unknown and there were no data to indicate that the changes in blood pressure did exceed the limits for clinical relevance. For these drugs, an alert for a DDSI was not considered relevant.

*Antidepressants*. Having depression is associated with increased blood pressure (Diminic-Lisica et al., 2014), and treating depression could lead to a reduction in blood pressure, despite the possible blood pressure-raising effect of antidepressants (Fu et al., 2015). In patients with hypertension, no risk of worsening hypertension was found for serotonin reuptake inhibitors (SSRIs) and venlafaxine (Thase, 1998; Diminic-Lisica et al., 2014; Fu et al., 2015; Razavi Ratki et al., 2016; Peixoto et al., 2019). In most patients using tricyclic antidepressants (TCAs), the increase in blood pressure did not reach the minimal clinically important difference (Licht et al., 2009; Breeden et al., 2018; Crookes et al., 2018).

#### 3.4.2 No evidence (data) for an effect of the drug on blood pressure

Antithrombotics and methylergometrine were evaluated because of the warning about hypertension in SmPC’s/Product information. However, we did not find studies that indicated that these drugs altered blood pressure. These agents are therefore not signaled for the DDSI hypertension.

For other drugs such disulfiram no warnings were stated in the product information, although there were case-reports that published increase in blood pressure. According to the expert panel, these anecdotal case-reports provide insufficient evidence to limit the use of these drugs in patients with hypertension.

#### 3.4.3 Short-term use

For the majority of blood pressure changes caused by ergotamine, imidazoline derivatives, prolactin inhibitors, sympathomimetics and triptans, the change in blood pressure is often transient. Due to the specific indication of drugs to cause transient high blood pressure or short-term use of some drugs, additional signaling through CDSSs was deemed not necessary for these drugs.

*Prolactin inhibitors*. Cases of intracranial hemorrhage and an increased risk of *postpartum* hypertension due to drug-induced hypertension have been reported for prolactin inhibitors (bromocriptine, cabergoline) (Watson et al., 1989; Kirsch et al., 2001). The manufacturers advise against the use of these drugs in hypertension, including gestational hypertension. However, based on available evidence and short-term use, the influence of prolactin inhibitors on blood pressure does not appear to be clinically relevant (Herings and Stricker, 1995; Gulleroglu et al., 2012; Bernard et al., 2015).

#### 3.4.4 Close monitoring of blood pressure according to clinical guidelines

Table 4 summarizes drugs of which the DDSI with hypertension required no further action because of close monitoring of blood pressure according to clinical guidelines (Lyon et al., 2022). For these drugs monitoring of blood pressure for all patients is already recommended in protocols, due to known influences on blood pressure. Since use of these drugs is safe because of regular monitoring of the blood pressure, additional signaling for a DDSI via CDSS were not considered relevant, even though a clinically relevant effect on blood pressure was found in literature. Furthermore, additional alerts for these drugs could lead to alert fatigue (van Tongeren et al., 2020).

*Calcineurin inhibitors*. In a systematic review and clinical studies, a mean increase in blood pressure of 11 mmHg was seen for high doses of cyclosporin, which were above the lower limit for clinically relevant increase of blood pressure (Taylor et al., 1999; Klein et al., 2002; Higgins et al., 2004; Snanoudj et al., 2004; Robert et al., 2010; Marienhagen et al., 2019). However, there did not appear to be a group effect here, as tacrolimus had minimal effect on blood pressure (Woo et al., 1997; Taylor et al., 1999; Klein et al., 2002). Moreover, transplantation, for which calcineurin inhibitors are often used, could also lead to hypertension itself (Hošková et al., 2017).

*MAO-inhibitors*. Hypertension has been reported as side effect for the use of monoamine oxidase inhibitors (MAOIs) (Keck et al., 1989; Lavin et al., 1993; Taylor et al., 2005), and therefore patients need to avoid tyramine-containing foods that might provoke hypertensive crisis. (Stahl and Felker, 2008; Stahl, 2013). Another common side effect of MAOIs is orthostatic hypotension, hence blood pressure is monitored for classical MAOIs (Zandee et al., 2017). As selective MAOIs (such as moclobemide) are hardly associated with hypertension, monitoring of blood pressure is not needed (Bonnet, 2003; Yamada and Yasuhara, 2004).

## 4 Discussion

While hypertension is mentioned as contra-indication in many sources including the official product information, only a few drugs have a clinically relevant DDSI with hypertension. Interactions with corticosteroids, danazol, and yohimbine are associated with a significant increase in blood pressure (more than 10 mmHg SBP or 5 mmHg DBP) and determined as clinically relevant by our expert panel. The majority of hypertension contra-indications mentioned in the SmPCs of drugs were determined to be not clinically relevant. The most common reasons for this were a minor influence on blood pressure, lack of data indicating an effect on blood pressure, and short-term use of the drug. Moreover, alerts for drugs used under close monitoring of blood pressure according to clinical guidelines, e.g., oncology protocols, were considered to be of no additional value for drug safety.

Our study reveals that for many drugs the statements of hypertension as a DDSI in product information are often poorly supported by data. Healthcare professionals might withhold first-choice medication based on these statements, which may lead to suboptimal treatment. To ensure that healthcare professionals make good decisions, product information should quantify blood pressure changes, if possible with expected effect on systolic or and diastolic values. This level of detail is already mandatory for other pharmacological information, such as drug-drug interactions that mention the effects on plasma levels and drug exposure (European Medicines Agencey EMA, 2009). In addition, product information should provide guidance on appropriate actions when a DDSI occurs, such as dose adjustments or blood pressure monitoring (Weersink et al., 2019). This knowledge should be developed in clinical studies investigating medication safety in patients with hypertension, leading to recommendations concerning discontinuation of treatment, dose adaptation, or monitoring of blood pressure.

While evidence indicates blood pressure changes for some drugs used by patients with specific conditions such as oncology or transplantation, additional DDSI alerts are considered unnecessary. This is because blood pressure is already closely monitored during therapy for these conditions according to clinical guidelines. Additional alerts would lead to alert fatigue and unnecessary contact about drug therapy between healthcare professionals (Phansalkar et al., 2013). Clinical guidelines may differ between countries and settings, resulting in varying local guidelines for DDSI in different settings and regions.

Since there are only statements in product information for the DDSI hypertension, recommendations for uncontrolled hypertension fall outside the scope of this study. Uncontrolled hypertension occurs when blood pressure remains high despite treatment. Reasons for this condition can be (pseudo-)resistance to antihypertensive drug treatment due to specific conditions such as transplantation, consumption of specific dietary ingredients (e.g., salt) or non-adherence to antihypertensives (Spence, 2018). It is important to identify the root cause of uncontrolled hypertension and to avoid drugs that can change blood pressure even further. For example, in women with uncontrolled hypertension, oral contraceptives should be avoided (ACOG Practice Bulletin, 2019; Shufelt and LeVee, 2020), whereas oral contraceptives are not signaled for hypertensive patients due to an on average minor influence on blood pressure. As uncontrolled hypertension is generally monitored intensively, a change in blood pressure due to drugs could be noticed earlier in patients with uncontrolled hypertension than in patients with controlled hypertension.

One of the most important strengths of this study is its unique approach in evaluating the interaction between hypertension and medication, providing practical recommendations following a combination of literature review and expert opinion. With limited studies available in patients with hypertension, expert opinion adds relevant pharmacological and clinical perspectives to the practical recommendations. Moreover, we examined all the drugs marketed in the Netherlands, representing the majority of the drugs available in other countries. The multidisciplinary character of the expert panel guaranteed that different perspectives were included and that the recommendations were applicable in a wide range of healthcare settings.

An additional strength of this approach is that this study contributes to limiting alert fatigue. Alert fatigue is one of the main reasons why alerts are overridden (van der Sijs et al., 2006; Isaac et al., 2009; Nanji et al., 2014; Shah et al., 2021). In a systematic review, the various factors influencing the efficiency and appropriateness of alerts in CDSSs was investigated. The review concluded that most of the alerts were not properly designed, which explained high frequency of alert overrides in clinical care (Olakotan and Mohd, 2021). To combat alert fatigue, our study contributes by suggesting only clinically relevant DDSIs. However, defining clinically relevant blood pressure change poses a challenge, necessitating a multidisciplinary approach. With this systematic methodology, it is possible to balance clinical relevance and alert fatigue, hereby preventing alert overrides and possible unsafe situations.

A limitation to this study is the absence of a clearly defined boundary for relevant changes in blood pressure. It is not possible to define what the expected effects of drugs would be on systolic and diastolic blood pressure prior to the evaluation. For this reason, we defined these boundaries in our multidisciplinary setting. However, the limits we used may be arbitrary, as there are specific patient groups that may require stricter monitoring. In some patient groups with specific comorbidities, even a smaller increase (>5 mmHg) in SBP may be important (Whelton et al., 2017). Moreover, the increase in SBP is more important in patients >50 years, and the increase in DBP is important in patients <50 years (Williams et al., 2018). Presumably, the risk for hypertension could increase when more risk factors are present. As the current DDSI-alerts do not involve additional risk factors, a future development could be that clinically important differences should be determined in the context of multiple individual patient characteristics.

A final limitation lies in the challenge of adopting our recommendations in settings in other countries. In the Netherlands, healthcare providers have the professional freedom to deviate from official documents such as the SmPC, which may be different in international settings (Eickhoff et al., 2021). In addition, our recommendations are based on Dutch treatment guidelines and practice. With this study, we aimed to contribute to the international dissemination and implementation of knowledge about DSSIs for hypertension.

This study identified a considerable knowledge gap for drugs that might have an influence on blood pressure, yet without sufficient clinical data to support that claim. Future studies should focus on the clinical relevance of these potential DDSIs and quantify the extend of blood pressure change. In countries where healthcare professionals can use data about the patient’s comorbidities in their electronic prescribing system, it is possible to integrate alerts in a CDSS (Diesveld et al., 2021). Implementation of automatic alerts in prescribing systems and CDSSs, combined with multidisciplinary cooperation, could enhance patient care and potentially reduce alert fatigue (Weissenborn et al., 2017). Future research should investigate the effectiveness of such implementations on safe drug use in patients with hypertension and reducing alert fatigue, time and costs.

## 5 Conclusion

With a systematic approach, we evaluated the interactions between prescribed drugs and hypertension. We developed practical recommendations for healthcare professionals, based on the available evidence and expert opinion and implemented these in clinical decision support systems in the Netherlands. These recommendations can serve as a basis to be applied in daily practice in international settings as well, to improve medication safety and patient safety.

## Data Availability

The datasets presented in this study can be found in online repositories. The names of the repository/repositories and accession number(s) can be found in the article/Supplementary Material.

## References

[B1] ACOG Practice Bulletin (2019). No. 206: Use of Hormonal Contraception in Women With Coexisting Medical Conditions. Obstetrics and Gynecology 133 (2), e128–50. 30681544 10.1097/AOG.0000000000003072

[B2] Baker J. F. Sauer B. Teng C. C. George M. Cannon G. W. Ibrahim S. (2018). Initiation of disease-modifying therapies in rheumatoid arthritis is associated with changes in blood pressure. JCR J. Clin. Rheumatology 24 (4), 203–209. 10.1097/RHU.0000000000000736 PMC7461421 29664818

[B3] Bernard N. Jantzem H. Becker M. Pecriaux C. Bénard-Laribière A. Montastruc J. (2015). Severe adverse effects of bromocriptine in lactation inhibition: a pharmacovigilance survey. BJOG 122 (9), 1244–1251. 10.1111/1471-0528.13352 25761676

[B4] Bloechliger M. Reinau D. Spoendlin J. Chang S. C. Kuhlbusch K. Heaney L. G. (2018). Adverse events profile of oral corticosteroids among asthma patients in the UK: cohort study with a nested case-control analysis. Respir. Res. 19 (1), 75. 10.1186/s12931-018-0742-y 29699563 PMC5921395

[B6] Bonnet U. (2003). Moclobemide: therapeutic use and clinical studies. CNS Drug Rev. 9 (1), 97–140. 10.1111/j.1527-3458.2003.tb00245.x 12595913 PMC6741704

[B7] Borgsteede S. D. Bogaard L. Diesveld M. M. E. van Donselaar - Pham T. K. L. Eimermann V. M. de Klerk S. (2023). Commentaren medicatiebewaking. Health Base Found.

[B8] Breeden M. Brieler J. Salas J. Scherrer J. F. (2018). Antidepressants and incident hypertension in primary care patients. J. Am. Board Fam. Med. 31 (1), 22–28. 10.3122/jabfm.2018.01.170234 29330236

[B9] Bretza J. A. Novey H. S. Vaziri N. D. Warner A. S. (1980). Hypertension: a complication of danazol therapy. Arch. Intern Med. 140 (10), 1379–1380. 10.1001/archinte.140.10.1379 7425772

[B10] Bundy J. D. Li C. Stuchlik P. Bu X. Kelly T. N. Mills K. T. (2017). Systolic blood pressure reduction and risk of cardiovascular disease and mortality: a systematic review and network meta-analysis. JAMA Cardiol. 2 (7), 775–781. 10.1001/jamacardio.2017.1421 28564682 PMC5710614

[B11] Centers for Disease Control and Prevention, National Center for Health Statistics (2022). About multiple cause of death, 1999–2020. CDC WONDER Online Database website. Atlanta, GA: Centers for Disease Control and Prevention.

[B12] Crookes D. M. Demmer R. T. Keyes K. M. Koenen K. C. Suglia S. F. (2018). Depressive symptoms, antidepressant use, and hypertension in young adulthood. Epidemiology 29 (4), 547–555. 10.1097/EDE.0000000000000840 29629939 PMC5980764

[B13] Damase-Michel C. Tran M. A. Llau M. E. Chollet F. Senard J. M. Guiraud-Chaumeil B. (1993). The effect of yohimbine on sympathetic responsiveness in essential hypertension. Eur. J. Clin. Pharmacol. 44 (2), 199–201. 10.1007/BF00315481 8453967

[B14] Diesveld M. M. E. de Klerk S. Cornu P. Strobach D. Taxis K. Borgsteede S. D. (2021). Management of drug-disease interactions: a best practice from The Netherlands. Int. J. Clin. Pharm. 43 (6), 1437–1450. 10.1007/s11096-021-01308-0 34273048

[B15] Diminic-Lisica I. Popovic B. Rebic J. Klaric M. Franciškovic T. (2014). Outcome of treatment with antidepressants in patients with hypertension and undetected depression. Int. J. Psychiatry Med. 47 (2), 115–129. 10.2190/PM.47.2.c 25084798

[B16] Distler A. Philipp T. Lüth B. Wucherer G. (1979). Studies on the mechanism of mineralocorticoid-induced blood pressure increase in man. Clin. Sci. 57 (s5), 303s–5s. 10.1042/cs057303s 396080

[B17] Eickhoff C. Griese-Mammen N. Mueller U. Said A. Schulz M. (2021). Primary healthcare policy and vision for community pharmacy and pharmacists in Germany. Pharm. Pract. (Granada) 19 (1), 2248. 10.18549/PharmPract.2021.1.2248 33520040 PMC7844970

[B18] Ettehad D. Emdin C. A. Kiran A. Anderson S. G. Callender T. Emberson J. (2016). Blood pressure lowering for prevention of cardiovascular disease and death: a systematic review and meta-analysis. Lancet 387 (10022), 957–967. 10.1016/S0140-6736(15)01225-8 26724178

[B19] European Medicines Agencey (EMA) (2009). A guideline on Summary of Product Characteristics (SmPC). European Commission, enterprise and industry directorate-general. Available at: https://health.ec.europa.eu/document/download/6a043dea-7d0f-4252-947b-cef58f53d37e_en.

[B20] Fardet L. Nazareth I. Petersen I. (2015). Synthetic glucocorticoids and early variations of blood pressure: a population-based cohort study. J. Clin. Endocrinol. Metab. 100 (7), 2777–2783. 10.1210/jc.2015-1127 25978108

[B21] Flint A. C. Conell C. Ren X. Banki N. M. Chan S. L. Rao V. A. (2019). Effect of systolic and diastolic blood pressure on cardiovascular outcomes. N. Engl. J. Med. 381 (3), 243–251. 10.1056/NEJMoa1803180 31314968

[B22] Fu W. Ma L. Zhao X. Li Y. Zhu H. Yang W. (2015). Antidepressant medication can improve hypertension in elderly patients with depression. J. Clin. Neurosci. 22 (12), 1911–1915. 10.1016/j.jocn.2015.03.067 26256065

[B23] Grossman E. Rosenthal T. Peleg E. Holmes C. Goldstein D. S. (1993). Oral yohimbine increases blood pressure and sympathetic nervous outflow in hypertensive patients. J. Cardiovasc Pharmacol. 22 (1), 22–26. 10.1097/00005344-199307000-00004 7690091

[B24] Gulleroglu K. Olgac A. Bayrakci U. Erdogan O. Kinik S. T. Baskin E. (2012). Hyperprolactinemia as a rare cause of hypertension in chronic renal failure. Ren. Fail 34 (6), 792–794. 10.3109/0886022X.2012.672313 22462393

[B25] Herings R. M. C. Stricker B. H. C. (1995). Bromocriptine and suppression of postpartum lactation. The incidence of adverse cardiovascular effects in women of child-bearing age. Pharm. World and Sci. 17 (4), 133–137. 10.1007/BF01872390 7581219

[B26] Higgins R. Ramaiyan K. Dasgupta T. Kanji H. Fletcher S. Lam F. (2004). Hyponatraemia and hyperkalaemia are more frequent in renal transplant recipients treated with tacrolimus than with cyclosporin. Further evidence for differences between cyclosporin and tacrolimus nephrotoxicities. Nephrol. Dial. Transplant. 19 (2), 444–450. 10.1093/ndt/gfg515 14736972

[B27] Hošková L. Málek I. Kopkan L. Kautzner J. (2017). Pathophysiological mechanisms of calcineurin inhibitor-induced nephrotoxicity and arterial hypertension. Physiological Res. 66, 167–180. 10.33549/physiolres.933332 27982677

[B28] Isaac T. Weissman J. S. Davis R. B. Massagli M. Cyrulik A. Sands D. Z. (2009). Overrides of medication alerts in ambulatory care. Arch. Intern Med. 169 (3), 305–311. 10.1001/archinternmed.2008.551 19204222

[B29] Keck P. E. Pope H. G. Nierenberg A. A. (1989). Autoinduction of hypertensive reactions by tranylcypromine? J. Clin. Psychopharmacol. 9 (1), 48–51. 10.1097/00004714-198902000-00011 2486182

[B30] Kirsch C. Iffy L. Zito G. E. McArdle J. J. (2001). The role of hypertension in bromocriptine-related puerperal intracranial hemorrhage. Neuroradiology 43 (4), 302–304. 10.1007/s002340000492 11338413

[B31] Klein IHHT Abrahams A. van Ede T. Hen?? R. J. Koomans H. A. Ligtenberg G. (2002). Different effects of tacrolimus and cyclosporine on renal hemodynamics and blood pressure in healthy subjects. Transplantation 73 (5), 732–736. 10.1097/00007890-200203150-00012 11907418

[B32] Lavin M. R. Mendelowitz A. Kronig M. H. (1993). Spontaneous hypertensive reactions with monoamine oxidase inhibitors. Biol. Psychiatry 34 (3), 146–151. 10.1016/0006-3223(93)90384-p 8399806

[B33] Licht C. M. M. de Geus E. J. C. Seldenrijk A. van Hout H. P. J. Zitman F. G. van Dyck R. (2009). Depression is associated with decreased blood pressure, but antidepressant use increases the risk for hypertension. Hypertension 53 (4), 631–638. 10.1161/HYPERTENSIONAHA.108.126698 19237679

[B34] Lyon A. R. López-Fernández T. Couch L. S. Asteggiano R. Aznar M. C. Bergler-Klein J. (2022). 2022 ESC guidelines on cardio-oncology developed in collaboration with the European hematology association (EHA), the European society for therapeutic radiology and oncology (ESTRO) and the international cardio-oncology society (IC-OS). Eur. Heart J. 43 (41), 4229–4361. 10.1093/eurheartj/ehac244 36017568

[B35] Mancia G. Kreutz R. Brunström M. Burnier M. Grassi G. Januszewicz A. (2023). 2023 ESH guidelines for the management of arterial hypertension the task force for the management of arterial hypertension of the European society of hypertension: endorsed by the international society of hypertension (ISH) and the European renal association (ERA). J. Hypertens. 41 (12), 1874–2071. 10.1097/HJH.0000000000003480 37345492

[B36] Marienhagen K. Lehner F. Klempnauer J. Hecker H. Borlak J. (2019). Treatment of cyclosporine induced hypertension: results from a long-term observational study using different antihypertensive medications. Vasc. Pharmacol. 115, 69–83. 10.1016/j.vph.2018.06.012 29933079

[B37] Miyabe Y. Takei T. Iwabuchi Y. Moriyama T. Nitta K. (2016). Amelioration of the adverse effects of prednisolone by rituximab treatment in adults with steroid-dependent minimal-change nephrotic syndrome. Clin. Exp. Nephrol. 20 (1), 103–110. 10.1007/s10157-015-1139-6 26138356

[B38] Musso N. R. Vergassola C. Pende A. Lotti G. (1995). Yohimbine effects on blood pressure and plasma catecholamines in human hypertension. Am. J. Hypertens. 8 (6), 565–571. 10.1016/0895-7061(95)00037-P 7662240

[B39] Nanji K. C. Slight S. P. Seger D. L. Cho I. Fiskio J. M. Redden L. M. (2014). Overrides of medication-related clinical decision support alerts in outpatients. J. Am. Med. Inf. Assoc. 21 (3), 487–491. 10.1136/amiajnl-2013-001813 PMC3994856 24166725

[B40] Olakotan O. O. Mohd Y. M. (2021). The appropriateness of clinical decision support systems alerts in supporting clinical workflows: a systematic review. Health Inf. J. 27 (2), 146045822110075. 10.1177/14604582211007536 33853395

[B41] Panoulas V. F. Douglas K. M. J. Stavropoulos-Kalinoglou A. Metsios G. S. Nightingale P. Kita M. D. (2008). Long-term exposure to medium-dose glucocorticoid therapy associates with hypertension in patients with rheumatoid arthritis. Rheumatology. 47 (1), 72–75. 10.1093/rheumatology/kem311 18077493

[B42] Pears J. Sandercock P. A. (1990). Benign intracranial hypertension associated with danazol. Scott Med. J. 35 (2), 49. 10.1177/003693309003500207 2374906

[B43] Peixoto M. Cesaretti M. Hood S. Tavares A. (2019). Effects of SSRI medication on heart rate and blood pressure in individuals with hypertension and depression. Clin. Exp. Hypertens. 41 (5), 428–433. 10.1080/10641963.2018.1501058 30047786

[B44] Phansalkar S. van der Sijs H. Tucker A. D. Desai A. A. Bell D. S. Teich J. M. (2013). Drug-drug interactions that should be non-interruptive in order to reduce alert fatigue in electronic health records. J. Am. Med. Inf. Assoc. 20 (3), 489–493. 10.1136/amiajnl-2012-001089 PMC3628052 23011124

[B45] Rapsomaniki E. Timmis A. George J. Pujades-Rodriguez M. Shah A. D. Denaxas S. (2014). Blood pressure and incidence of twelve cardiovascular diseases: lifetime risks, healthy life-years lost, and age-specific associations in 1·25 million people. Lancet 383 (9932), 1899–1911. 10.1016/S0140-6736(14)60685-1 24881994 PMC4042017

[B46] Razavi Ratki S. K. Seyedhosseini S. Valizadeh A. Rastgoo T. Tavakkoli R. Golabchi A. (2016). Can antidepressant drug impact on blood pressure level in patients with psychiatric disorder and hypertension? A randomized trial. Int. J. Prev. Med. 7 (1), 26. 10.4103/2008-7802.174891 26941927 PMC4755252

[B47] Rice J. B. White A. G. Johnson M. Wagh A. Qin Y. Bartels-Peculis L. (2018). Quantitative characterization of the relationship between levels of extended corticosteroid use and related adverse events in a US population. Curr. Med. Res. Opin. 34 (8), 1519–1527. 10.1080/03007995.2018.1474090 29741130

[B48] Robert N. Wong G. W. Wright J. M. (2010). Effect of cyclosporine on blood pressure. Cochrane Database Syst. Rev., CD007893. 10.1002/14651858.CD007893.pub2 20091657 PMC12665455

[B49] Royal Dutch Pharmacists Association Koninklijke Nederlandse Maatschappij ter bevordering der Pharmacie (2023). Medicines information centre. Contra-Indicatie aandoeningen. Available at: https://kennisbank.knmp.nl .

[B50] Samadian F. Dalili N. Jamalian A. (2016). Lifestyle modifications to prevent and control hypertension. Iran. J. Kidney Dis. 10 (5), 237–263. 27721223

[B51] Sazliyana S. Mohd Shahrir M. Kong C. N. Tan H. Hamidon B. Azmi M. (2011). Implications of immunosuppressive agents in cardiovascular risks and carotid intima media thickness among lupus nephritis patients. Lupus. 20 (12), 1260–1266. 10.1177/0961203311411347 21844115

[B52] Shah S. N. Amato M. G. Garlo K. G. Seger D. L. Bates D. W. (2021). Renal medication-related clinical decision support (CDS) alerts and overrides in the inpatient setting following implementation of a commercial electronic health record: implications for designing more effective alerts. J. Am. Med. Inf. Assoc. 28 (6), 1081–1087. 10.1093/jamia/ocaa222 PMC8661393 33517413

[B53] Shufelt C. LeVee A. (2020). Hormonal contraception in women with hypertension. JAMA 324 (14), 1451–1452. 10.1001/jama.2020.11935 32955577 PMC8528006

[B54] Snanoudj R. Kriaa F. Arzouk N. Beaudreuil S. Hiesse C. Durrbach A. (2004). Single-Center experience with cyclosporine therapy for kidney transplantation: analysis of a Twenty-Year period in 1200 patients. Transpl. Proc. 36 (2), 83S–8. 10.1016/j.transproceed.2004.01.089 15041313

[B55] Song P. Zhang Y. Yu J. Zha M. Zhu Y. Rahimi K. (2019). Global prevalence of hypertension in children: a systematic review and meta-analysis. JAMA Pediatr. 173 (12), 1154–1163. 10.1001/jamapediatrics.2019.3310 31589252 PMC6784751

[B56] Spence J. D. (2018). Controlling resistant hypertension. Stroke Vasc. Neurol. 3 (2), 69–75. 10.1136/svn-2017-000138 30022799 PMC6047342

[B57] Stahl S. M. (2013). “Essential psychopharmacology,” in Neuroscientific basis and practical applications. 4 (Cambridge: Cambridge Medicine).

[B58] Stahl S. M. Felker A. (2008). Monoamine oxidase inhibitors: *a modern Guide to an unrequited Class of antidepressants* . CNS Spectr. 13 (10), 855–870. 10.1017/s1092852900016965 18955941

[B59] Taylor B. P. Quitkin F. M. McGrath P. J. Stewart J. W. (2005). Do antihypertensives make tranylcypromine safer? J. Clin. Psychiatry 66 (05), 657–658. 10.4088/jcp.v66n0519e 15889959

[B60] Taylor D. O. Barr M. L. Radovancevic B. Renlund D. G. Mentzer Jr R. M. Smart F. W. (1999). A randomized, multicenter comparison of tacrolimus and cyclosporine immunosuppressive regimens in cardiac transplantation: decreased hyperlipidemia and hypertension with tacrolimus. J. Heart Lung Transplant. 18 (4), 336–345. 10.1016/s1053-2498(98)00060-6 10226898

[B61] Thase M. E. (1998). Effects of venlafaxine on blood pressure: a meta-analysis of original data from 3744 depressed patients. J. Clin. Psychiatry 59 (10), 502–508. 10.4088/jcp.v59n1002 9818630

[B62] van der Sijs H. Aarts J. Vulto A. Berg M. (2006). Overriding of drug safety alerts in computerized physician order entry. J. Am. Med. Inf. Assoc. 13 (2), 138–147. 10.1197/jamia.M1809 PMC1447540 16357358

[B63] van Roon E. N. Flikweert S. le Comte M. Langendijk P. N. J. Kwee-Zuiderwijk W. J. M. Smits P. (2005). Clinical relevance of drug-drug interactions: a structured assessment procedure. Drug Saf. 28 (12), 1131–1139. 10.2165/00002018-200528120-00007 16329715

[B64] van Tongeren J. M. Z. Harkes-Idzinga S. F. van der Sijs H. Atiqi R. van den Bemt B. J. F. Draijer L. W. (2020). The development of practice recommendations for drug-disease interactions by literature review and expert opinion. Front. Pharmacol. 11, 707. 10.3389/fphar.2020.00707 32499701 PMC7243438

[B65] Vishram J. K. K. Borglykke A. Andreasen A. H. Jeppesen J. Ibsen H. Jørgensen T. (2012). Impact of age on the importance of systolic and diastolic blood pressures for stroke risk: the MOnica, Risk, Genetics, Archiving, and Monograph (MORGAM) Project. Hypertension 60 (5), 1117–1123. 10.1161/HYPERTENSIONAHA.112.201400 23006731

[B66] Volksgezondheid en Zorg info (2022). Ziekten van hart en vaatstelsel Sterftecijfers. Available at: https://www.vzinfo.nl/hart-en-vaatziekten/sterftecijfers .

[B67] Watson D. L. Bhatia R. K. Norman G. S. Brindley B. A. Sokol R. J. (1989). Bromocriptine mesylate for lactation suppression: a risk for postpartum hypertension? Obstetrics Gynecol. 74 (4), 573–576. 2797633

[B68] Weersink R. A. Timmermans L. Monster-Simons M. H. Mol P. G. M. Metselaar H. J. Borgsteede S. D. (2019). Evaluation of information in summaries of product characteristics (SmPCs) on the use of a medicine in patients with hepatic impairment. Front. Pharmacol. 10, 1031. 10.3389/fphar.2019.01031 31607904 PMC6758592

[B69] Weissenborn M. Haefeli W. E. Peters-Klimm F. Seidling H. M. (2017). Interprofessional communication between community pharmacists and general practitioners: a qualitative study. Int. J. Clin. Pharm. 39 (3), 495–506. 10.1007/s11096-017-0450-6 28315115

[B70] Whelton P. K. Carey R. M. Aronow W. S. Casey D. E. Collins K. J. Dennison Himmelfarb C. (2017). 2017 ACC/AHA/AAPA/ABC/ACPM/AGS/APhA/ASH/ASPC/NMA/PCNA guideline for the prevention, detection, evaluation, and management of high blood pressure in adults: a report of the American College of Cardiology/American heart association task force on clinical practice guidelines. Hypertension 71 (6), e13–e115. 10.1161/HYP.0000000000000065 29133356

[B71] Whelton P. K. Carey R. M. Mancia G. Kreutz R. Bundy J. D. Williams B. (2022). Harmonization of the American College of Cardiology/American heart association and European society of Cardiology/European society of hypertension blood pressure/hypertension guidelines: comparisons, reflections, and recommendations. J. Am. Coll. Cardiol. 80 (12), 1192–1201. 10.1016/j.jacc.2022.07.005 35965201

[B72] Williams B. Mancia G. Spiering W. Agabiti Rosei E. Azizi M. Burnier M. (2018). 2018 practice guidelines for the management of arterial hypertension of the European society of Cardiology and the European society of hypertension. Blood Press 27 (6), 314–340. 10.1080/08037051.2018.1527177 30380928

[B73] Woo M. Przepiorka D. Ippoliti C. Warkentin D. Khouri I. Fritsche H. (1997). Toxicities of tacrolimus and cyclosporin A after allogeneic blood stem cell transplantation. Bone Marrow Transpl. 20 (12), 1095–1098. 10.1038/sj.bmt.1701027 9466284

[B74] Yamada M. Yasuhara H. (2004). Clinical pharmacology of MAO inhibitors: safety and future. Neurotoxicology 25 (1–2), 215–221. 10.1016/S0161-813X(03)00097-4 14697896

[B75] Zandee W. T. Alsma J. Birkenhäger T. K. den Meiracker A. H. V. A. N. van Hoek M. Versmissen J. (2017). Hypertension en orthostatic hypotension during use of monoamine oxidase (MAO) inhibitors. Tijdschr. Psychiatr. 59 (6), 366–371. 28613369

