# Supplementary material for: Developing practical recommendations for drug-disease interactions in patients with hypertension
Source: Front Pharmacol. 2024 Apr 17;15:1360146. doi: 10.3389/fphar.2024.1360146 (PMC11061388; doi:10.3389/fphar.2024.1360146)
Supplement: Supplementary file 1 [file DataSheet1.pdf]

Supplementary Material

Developing practical recommendations for drug-disease interactions in patients with hypertension

Kübra Özokcu[1,2], Maaike M. E. Diesveld[3], Suzan G.H. Gipmans[4], Laura E. J. Peeters[5], Bert-Jan van den Born[6], Sander D. Borgsteede[3*]

[1]Department of Hospital Pharmacy, Meander Medisch Centrum, Amersfoort, the Netherlands

[2]Department of Hospital Pharmacy, Ziekenhuis Rivierenland, Tiel, the Netherlands

[3]Department of Clinical Decision Support, Healthbase Foundation, Houten, the Netherlands

[4]Medicines Information Centre, Royal Dutch Pharmacists Association (KNMP), The Hague, the Netherlands

[5]Department of Hospital Pharmacy, Maasstad Hospital, Rotterdam, the Netherlands

[6]Departments of Internal Medicine and Public Health Amsterdam Cardiovascular Sciences Amsterdam UMC, Location AMC, Amsterdam, the Netherlands

*** Correspondence:**
Sander D. Borgsteede
sander.borgsteede@healthbase.nl

1 Supplementary Data – References of table 2, table 3 and table 4

1. McKay LI, Cidlowski JA. Physiologic and Pharmacologic Effects of Corticosteroids. Holland-Frei Cancer Medicine 6th edition Hamilton (ON): BC Decker; 2003.

2. Prednisolone 20 mg tablets SmPC. 11-05-2021. Available from: https://www.geneesmiddeleninformatiebank.nl/smpc/h105828_smpc.pdf.

3. Prednisolone 5 mg tablets. Prescribing Information - Food and Drug Administration. 21/03/2023. Available from: https://nctr-crs.fda.gov/fdalabel/services/spl/set-ids/070f1937-50a5-457f-bef5-4e597014e26d/spl-doc?hl=prednisolone .

4. Baker JF, Sauer B, Teng CC, George M, Cannon GW, Ibrahim S, et al. Initiation of Disease-Modifying Therapies in Rheumatoid Arthritis Is Associated With Changes in Blood Pressure. JCR: Journal of Clinical Rheumatology. 2018 Jun;24(4):203–9.

5. Bloechliger M, Reinau D, Spoendlin J, Chang SC, Kuhlbusch K, Heaney LG, et al. Adverse events profile of oral corticosteroids among asthma patients in the UK: cohort study with a nested case-control analysis. Respir Res. 2018 Dec 27;19(1):75.

6. Rice JB, White AG, Johnson M, Wagh A, Qin Y, Bartels-Peculis L, et al. Quantitative characterization of the relationship between levels of extended corticosteroid use and related adverse events in a US population. Curr Med Res Opin. 2018 Aug 3;34(8):1519–27.

7. Miyabe Y, Takei T, Iwabuchi Y, Moriyama T, Nitta K. Amelioration of the adverse effects of prednisolone by rituximab treatment in adults with steroid-dependent minimal-change nephrotic syndrome. Clin Exp Nephrol. 2016 Feb 3;20(1):103–10.

8. Fardet L, Nazareth I, Petersen I. Synthetic Glucocorticoids and Early Variations of Blood Pressure: A Population-Based Cohort Study. J Clin Endocrinol Metab. 2015 Jul;100(7):2777–83.

9. Sazliyana S, Mohd Shahrir M, Kong CN, Tan H, Hamidon B, Azmi M. Implications of immunosuppressive agents in cardiovascular risks and carotid intima media thickness among lupus nephritis patients. Lupus. 2011 Oct 15;20(12):1260–6.

10. Panoulas VF, Douglas KMJ, Stavropoulos-Kalinoglou A, Metsios GS, Nightingale P, Kita MD, et al. Long-term exposure to medium-dose glucocorticoid therapy associates with hypertension in patients with rheumatoid arthritis. Rheumatology. 2008 Jan 1;47(1):72–5.

11. Distler A, Philipp T, Lüth B, Wucherer G. Studies on the Mechanism of Mineralocorticoid-Induced Blood Pressure Increase in Man. Clin Sci. 1979 Dec 1;57(s5):303s–5s.

12. Beck KR, Thompson GR, Odermatt A. Drug-induced endocrine blood pressure elevation. Pharmacol Res. 2020 Apr;154:104311.

13. Danazol 100 mg capsules SmPC. 25-04-2021. Available from: https://www.geneesmiddeleninformatiebank.nl/smpc/h06982_smpc.pdf .

14. Danazol 50 mg, 100 mg or 200 mg capsules. Prescribing Information - Food and Drug Administration. 03/03/2023. Available from: https://nctr-crs.fda.gov/fdalabel/services/spl/set-ids/55ad6325-16f6-4f0c-a1dc-734847052d0b/spl-doc?hl=danazol .

15. Pears J, Sandercock PA. Benign Intracranial Hypertension Associated with Danazol. Scott Med J. 1990 Apr 25;35(2):49–49.

16. Bretza JA. Hypertension: a complication of danazol therapy. Arch Intern Med. 1980 Oct 1;140(10):1379–80.

17. Musso NR, Vergassola C, Pende A, Lotti G. Yohimbine effects on blood pressure and plasma catecholamines in human hypertension*. Am J Hypertens. 1995 Jun;8(6):565–71.

18. Damase-Michel C, Tran MA, Llau ME, Chollet F, Senard JM, Guiraud-Chaumeil B, et al. The effect of yohimbine on sympathetic responsiveness in essential hypertension. Eur J Clin Pharmacol. 1993;44(2):199–201.

19. Grossman E, Rosenthal T, Peleg E, Holmes C, Goldstein DS. Oral Yohimbine Increases Blood Pressure and Sympathetic Nervous Outflow in Hypertensive Patients. J Cardiovasc Pharmacol. 1993 Jul;22(1):22–6.

20. Yohimbine 5 mg tablets SmPC. 12-2018. Available from: https://www.bcfi.be/nl/chapters/8?frag=6346&view=pvt&vmp_group=32342 .

21. Volkow ND, Wang GJ, Fowler JS, Molina PE, Logan J, Gatley SJ, et al. Cardiovascular effects of methylphenidate in humans are associated with increases of dopamine in brain and of epinephrine in plasma. Psychopharmacology (Berl). 2003 Mar 13;166(3):264–70.

22. Dexamfetamine, Tentin®. Summary of Product Characteristics. CBG Geneesmiddeleninformatiebank. 19/08/2022. Available from: https://www.geneesmiddeleninformatiebank.nl/smpc/h126113_smpc.pdf.

23. Lisdexamfetamine. Prescribing Information. Food and Drug Administration. 12/07/2023. Available from: https://nctr-crs.fda.gov/fdalabel/services/spl/set-ids/704e4378-ca83-445c-8b45-3cfa51c1ecad/spl-doc?hl=vyvanse.

24. Busold-Hagenbeck D, Elmenhorst J, Irtel von Brenndorff C, Hilgers R, Hulpke-Wette M. Frequency and individual severity of arterial blood pressure changes in children and adolescents with attention-deficit/hyperactivity disorder treated with methylphenidate hydrochloride: a prospective non-interventional study. Gen Psychiatr. 2020 Apr 26;33(2):e100193.

25. Liang EF, Lim SZ, Tam WW, Ho CS, Zhang MW, McIntyre RS, et al. The Effect of Methylphenidate and Atomoxetine on Heart Rate and Systolic Blood Pressure in Young People and Adults with Attention-Deficit Hyperactivity Disorder (ADHD): Systematic Review, Meta-Analysis, and Meta-Regression. Int J Environ Res Public Health. 2018 Aug 20;15(8):1789.

26. Hennissen L, Bakker MJ, Banaschewski T, Carucci S, Coghill D, Danckaerts M, et al. Cardiovascular Effects of Stimulant and Non-Stimulant Medication for Children and Adolescents with ADHD: A Systematic Review and Meta-Analysis of Trials of Methylphenidate, Amphetamines and Atomoxetine. CNS Drugs. 2017 Mar 24;31(3):199–215.

27. Awudu GAH, Besag FMC. Cardiovascular Effects of Methylphenidate, Amphetamines and Atomoxetine in the Treatment of Attention-Deficit Hyperactivity Disorder: An Update. Drug Saf. 2014 Sep 16;37(9):661–76.

28. Mick E, McManus DD, Goldberg RJ. Meta-analysis of increased heart rate and blood pressure associated with CNS stimulant treatment of ADHD in adults. European Neuropsychopharmacology. 2013 Jun;23(6):534–41.

29. Grace F, Sculthorpe N, Baker J, Davies B. Blood pressure and rate pressure product response in males using high-dose anabolic androgenic steroids (AAS). J Sci Med Sport. 2003 Sep;6(3):307–12.

30. Kienitz T, Quinkler M. Testosterone and Blood Pressure Regulation. Kidney Blood Press Res. 2008;31(2):71–9.

31. Ruige JB, Ouwens DM, Kaufman JM. Beneficial and Adverse Effects of Testosterone on the Cardiovascular System in Men. J Clin Endocrinol Metab. 2013 Nov 1;98(11):4300–10.

32. Barbosa Neto O, da Mota GR, de Sordi CC, Resende EAMR, Resende LAPR, Vieira da Silva MA, et al. Long-term anabolic steroids in male bodybuilders induce cardiovascular structural and autonomic abnormalities. Clinical Autonomic Research. 2018 Apr 10;28(2):231–44.

33. Liu JD, Wu YQ. Anabolic-androgenic steroids and cardiovascular risk. Chin Med J (Engl). 2019 Sep 20;132(18):2229–36.

34. Testosteron, Androgel®. Summary of Product Characteristics. CBG Geneesmiddeleninformatiebank. 03/02/2021. Available from: https://www.geneesmiddeleninformatiebank.nl/smpc/h115746_smpc.pdf.

35. Testosterone undecanoate, Kyzatrex®. Prescribing Information. Food and Drug Administration. 18/10/2022. Available from: https://nctr-crs.fda.gov/fdalabel/services/spl/set-ids/7f7167a7-2a25-47e2-acf5-33f499fce971/spl-doc?hl=kyzatrex.

36. Islam RM, Bell RJ, Green S, Page MJ, Davis SR. Safety and efficacy of testosterone for women: a systematic review and meta-analysis of randomised controlled trial data. Lancet Diabetes Endocrinol. 2019 Oct;7(10):754–66.

37. Corona G, Giagulli VA, Maseroli E, Vignozzi L, Aversa A, Zitzmann M, et al. Testosterone supplementation and body composition: results from a meta-analysis of observational studies. J Endocrinol Invest. 2016 Sep 30;39(9):967–81.

38. Corona G, Monami M, Rastrelli G, Aversa A, Sforza A, Lenzi A, et al. Type 2 diabetes mellitus and testosterone: a meta-analysis study. Int J Androl. 2011 Dec;34(6pt1):528–40.

39. Fernández-Balsells MM, Murad MH, Lane M, Lampropulos JF, Albuquerque F, Mullan RJ, et al. Adverse Effects of Testosterone Therapy in Adult Men: A Systematic Review and Meta-Analysis. J Clin Endocrinol Metab. 2010 Jun;95(6):2560–75.

40. Basaria S, Coviello AD, Travison TG, Storer TW, Farwell WR, Jette AM, et al. Adverse Events Associated with Testosterone Administration. New England Journal of Medicine. 2010 Jul 8;363(2):109–22.

41. Boxer RS, Kleppinger A, Brindisi J, Feinn R, Burleson JA, Kenny AM. Effects of dehydroepiandrosterone (DHEA) on cardiovascular risk factors in older women with frailty characteristics. Age Ageing. 2010 Jul 1;39(4):451–8.

42. Haddad RM, Kennedy CC, Caples SM, Tracz MJ, Boloña ER, Sideras K, et al. Testosterone and Cardiovascular Risk in Men: A Systematic Review and Meta-analysis of Randomized Placebo-Controlled Trials. Mayo Clin Proc. 2007 Jan;82(1):29–39.

43. Wang F, He Y, O. Santos H, Sathian B, C. Price J, Diao J. The effects of dehydroepiandrosterone (DHEA) supplementation on body composition and blood pressure: A meta-analysis of randomized clinical trials. Steroids. 2020 Nov;163:108710.

44. Gómez-Santos C, Hernández-Morante JJ, Tébar FJ, Granero E, Garaulet M. Differential effect of oral dehydroepiandrosterone-sulphate on metabolic syndrome features in pre- and postmenopausal obese women. Clin Endocrinol (Oxf). 2012 Oct;77(4):548–54.

45. Fu W, Ma L, Zhao X, Li Y, Zhu H, Yang W, et al. Antidepressant medication can improve hypertension in elderly patients with depression. Journal of Clinical Neuroscience. 2015 Dec;22(12):1911–5.

46. Haelst IMM v., Klei WA v., Doodeman HJ, Kalkman CJ, Egberts TCG. Selective Serotonin Reuptake Inhibitors and Intraoperative Blood Pressure. Am J Hypertens. 2012 Feb 1;25(2):223–8.

47. Nortriptyline, Nortrilen®. Summary of Product Characteristics. CBG Geneesmiddeleninformatiebank. 11/05/2022. Available from: https://www.geneesmiddeleninformatiebank.nl/smpc/h03285_smpc.pdf.

48. Citalopram, Cipramil®. Summary of Product Characteristics. CBG Geneesmiddeleninformatiebank; 20/05/2022. Available from: https://www.geneesmiddeleninformatiebank.nl/smpc/h19593_smpc.pdf .

49. Duloxetine, Cymbalta®. Product Information. European Medicines Agency (EN); 21-12-2021. Available from: https://www.ema.europa.eu/en/documents/product-information/cymbalta-epar-product-information_en.pdf .

50. Nortriptyline hydrochloride. Prescribing Information. Food and Drug Administration; 26-06-2023. Available from: https://nctr-crs.fda.gov/fdalabel/services/spl/set-ids/b0a7c4b8-973a-4ef3-ab91-0080e46a181f/spl-doc?hl=nortriptyline.

51. Citalopram. Prescribing Information. Food and Drug Administration; 17-07-2023. Available from: https://nctr-crs.fda.gov/fdalabel/services/spl/set-ids/b1f0c6e8-85eb-4368-8709-9cace32415cb/spl-doc?hl=citalopram.

52. Duloxetine. Prescribing Information. Food and Drug Administration. 20-07-2023. Available from: https://nctr-crs.fda.gov/fdalabel/services/spl/set-ids/2be9e5fb-011e-424b-b504-df2a28b5271d/spl-doc?hl=duloxetine .

53. Breeden M, Brieler J, Salas J, Scherrer JF. Antidepressants and Incident Hypertension in Primary Care Patients. The Journal of the American Board of Family Medicine. 2018 Jan 12;31(1):22–8.

54. Crookes DM, Demmer RT, Keyes KM, Koenen KC, Suglia SF. Depressive Symptoms, Antidepressant Use, and Hypertension in Young Adulthood. Epidemiology. 2018 Jul;29(4):547–55.

55. Licht CMM, de Geus EJC, Seldenrijk A, van Hout HPJ, Zitman FG, van Dyck R, et al. Depression Is Associated With Decreased Blood Pressure, but Antidepressant Use Increases the Risk for Hypertension. Hypertension. 2009 Apr;53(4):631–8.

56. Diminic-Lisica I, Popovic B, Rebic J, Klaric M, Franciškovic T. Outcome of Treatment with Antidepressants in Patients with Hypertension and Undetected Depression. The International Journal of Psychiatry in Medicine. 2014 Feb 30;47(2):115–29.

57. Peixoto M, Cesaretti M, Hood S, Tavares A. Effects of SSRI medication on heart rate and blood pressure in individuals with hypertension and depression. Clin Exp Hypertens. 2019 Jul 4;41(5):428–33.

58. Razavi Ratki SK, Seyedhosseini S, Valizadeh A, Rastgoo T, Tavakkoli R, Golabchi A, et al. Can antidepressant drug impact on blood pressure level in patients with psychiatric disorder and hypertension? A randomized trial. Int J Prev Med. 2016;7(1):26.

59. Thase ME. Effects of Venlafaxine on Blood Pressure. J Clin Psychiatry. 1998 Oct 15;59(10):502–8.

60. Wernicke JF, Prakash A, Kajdasz DK, Houston J. Safety and tolerability of duloxetine treatment of diabetic peripheral neuropathic pain between patients with and without cardiovascular conditions. J Diabetes Complications. 2009 Sep;23(5):349–59.

61. Wernicke JF, Faries D, Girod D, Brown JW, Gao H, Kelsey D, et al. Cardiovascular Effects of Atomoxetine in Children, Adolescents, and Adults. Drug Saf. 2003;26(10):729–40.

62. Atomoxetine. Summary of Product Characteristics. CBG Geneesmiddeleninformatiebank; 22/09/2022. Available from: https://www.geneesmiddeleninformatiebank.nl/smpc/h120972_smpc_en.pdf .

63. Atomoxetine, Strattera®. Prescribing Information. Food and Drug Administration. 06/01/2022. Available from: https://nctr-crs.fda.gov/fdalabel/services/spl/set-ids/309de576-c318-404a-bc15-660c2b1876fb/spl-doc?hl=strattera .

64. Sofuoglu M, Sewell RA. Norepinephrine and stimulant addiction. Addiction Biology. 2009 Apr;14(2):119–29.

65. Kulkarni RR, Ramdurg SI, Bairy BK. Disulfiram-Induced Reversible Hypertension: A Prospective Case Series and Review of The Literature. Indian J Psychol Med. 2014 Oct 1;36(4):434–8.

66. Zapata E, Orwin A. Severe hypertension and bronchospasm during disulfiram-ethanol test reaction. BMJ. 1992 Oct 10;305(6858):870–870.

67. Silver DF, Ewing JA, Rouse BA, Mueller RA. Responses to Disulfiram in Healthy Young Men; a Double-Blind Study. J Stud Alcohol. 1979 Nov;40(11):1003–13.

68. Grossman E. High blood pressure. A side effect of drugs, poisons, and food. Arch Intern Med. 1995 Mar 13;155(5):450–60.

69. Desogestrel-ethynilestradiol, Bryoronna®. Summary of Product Characteristics. CBG Geneesmiddeleninformatiebank; 26-10-2022. Available from: https://www.geneesmiddeleninformatiebank.nl/smpc/h110874_smpc.pdf .

70. Desogestrel-ethinylestradiol, Velivet®. Prescribing Information. Food and Drug Administration. 06/07/2023. Available from: https://nctr-crs.fda.gov/fdalabel/services/spl/set-ids/e7f11ebb-365e-4a31-bf36-6e924251a530/spl-doc?hl=velivet .

71. Park H, Kim K. Associations between oral contraceptive use and risks of hypertension and prehypertension in a cross-sectional study of Korean women. BMC Womens Health. 2013 Dec 21;13(1):39.

72. Cagnacci A, Zanin R, Napolitano A, Arangino S, Volpe A. Modification of 24-h ambulatory blood pressure and heart rate during contraception with the vaginal ring: A prospective study. Contraception. 2013 Oct;88(4):539–43.

73. Clyburn EB, DiPette DJ. Hypertension induced by drugs and other substances. Vol. 15, Seminars in Nephrology. 1995. p. 72–86.

74. Metyrapon, Metopirone®. Summary of Product Characteristics. CBG Geneesmiddeleninformatiebank; 13-04-2022. Available from: https://www.geneesmiddeleninformatiebank.nl/smpc/h113510_smpc.pdf .

75. Metyrapon, Metopirone®. Prescribing Information. Food and Drug Administration. 23/02/2023. Available from: https://nctr-crs.fda.gov/fdalabel/services/spl/set-ids/3a27d0ef-83a7-4e1c-9430-46a3326ee8d8/spl-doc?hl=metopirone .

76. Vögelin M, Cathomas R, Kamber N, Fehr T. Hypokalaemic metabolic alkalosis, hypertension and diabetes: what is the link. BMJ Case Rep. 2019 Jan 18;12(1):bcr-2018-227068.

77. Loh RKC, Formosa MF, La Gerche A, Reutens AT, Kingwell BA, Carey AL. Acute metabolic and cardiovascular effects of mirabegron in healthy individuals. Diabetes Obes Metab. 2019 Feb;21(2):276–84.

78. Mirabegron, Betmiga®. Product Information. European Medicines Agency (EN); 05-11-2021. Available from: https://www.ema.europa.eu/en/documents/product-information/betmiga-epar-product-information_en.pdf .

79. Mirabegron, Myrbetriq®. Prescribing Information. Food and Drug Administration. 15/12/2022. Available from: https://nctr-crs.fda.gov/fdalabel/services/spl/set-ids/aaaf3bea-1b21-4edf-9300-b7159ea9ede8/spl-doc?hl=myrbetriq .

80. Wang J, Zhou Z, Cui Y, Li Y, Yuan H, Gao Z, et al. Meta-analysis of the efficacy and safety of mirabegron and solifenacin monotherapy for overactive bladder. Neurourol Urodyn. 2019 Jan;38(1):22–30.

81. Chen HL, Chen TC, Chang HM, Juan YS, Huang WH, Pan HF, et al. Mirabegron is alternative to antimuscarinic agents for overactive bladder without higher risk in hypertension: a systematic review and meta-analysis. World J Urol. 2018 Aug 19;36(8):1285–97.

82. Sebastianelli A, Russo GI, Kaplan SA, McVary KT, Moncada I, Gravas S, et al. Systematic review and meta-analysis on the efficacy and tolerability of mirabegron for the treatment of storage lower urinary tract symptoms/overactive bladder: Comparison with placebo and tolterodine. International Journal of Urology. 2018 Mar;25(3):196–205.

83. White WB, Siddiqui E, Tat T, Franks B, Schermer CR. Cardiovascular safety of mirabegron: analysis of an integrated clinical trial database of patients with overactive bladder syndrome. Journal of the American Society of Hypertension. 2018 Nov;12(11):768-778.e1.

84. Pope JE, Anderson JJ, Felson DT. A meta-analysis of the effects of nonsteroidal anti-inflammatory drugs on blood pressure. Arch Intern Med. 1993 Feb 22;153(4):477–84.

85. Naproxen, Aleve®. Summary of Product Characteristics. CBG Geneesmiddeleninformatiebank; 23-11-2022. Available from: https://www.geneesmiddeleninformatiebank.nl/smpc/h19630_smpc.pdf .

86. Naproxen. Prescribing Information. Food and Drug Administration. 18/07/2023. Available from: https://nctr-crs.fda.gov/fdalabel/services/spl/set-ids/1acfb8cc-6faf-4b3e-9304-4819c42de0a6/spl-doc?hl=naproxen .

87. Ruoff GE. The impact of nonsteroidal anti-inflammatory drugs on hypertension: alternative analgesics for patients at risk. Clin Ther. 1998 May;20(3):376–87.

88. Chan CC, Reid CM, Aw TJ, Liew D, Haas SJ, Krum H. Do COX-2 inhibitors raise blood pressure more than nonselective NSAIDs and placebo? An updated meta-analysis. J Hypertens. 2009 Dec;27(12):2332–41.

89. Morrison A, Rosen Ramey D, van Adelsberg J, Watson DJ. Systematic review of trials of the effect of continued use of oral non-selective NSAIDs on blood pressure and hypertension. Curr Med Res Opin. 2007 Oct 21;23(10):2395–404.

90. Michibayashi T. Inhibitory action of prostaglandin E1 on smooth muscle contraction and calcium responses. Prostaglandins. 1978 May;15(5):803–12.

91. Misoprostol, Cytotec®. Prescribing Information. Food and Drug Administration. 09/07/2021. Available from: https://nctr-crs.fda.gov/fdalabel/services/spl/set-ids/4ab12da7-5731-4e06-bf1c-bc3f2e711f12/spl-doc?hl=cytotec .

92. Michibayashi T. Mechanism of Action of Hypotensive Prostaglandins in Patients with Essential Hypertension. Journal of Smooth Muscle Research. 2002;38(3):51–61.

93. Akkuzu B, Yilmaz I, Cakmak O, Ozluoglu LN. Efficacy of misoprostol in the treatment of tinnitus in patients with diabetes and/or hypertension. Auris Nasus Larynx. 2004 Sep;31(3):226–32.

94. Blankfield RP. Blood pressure, fluid retention and the cardiovascular risk of drugs. Future Cardiol. 2012 Jul;8(4):489–93.

95. Lo C, Toyama T, Wang Y, Lin J, Hirakawa Y, Jun M, et al. Insulin and glucose-lowering agents for treating people with diabetes and chronic kidney disease. Cochrane Database of Systematic Reviews. 2018 Sep 24;

96. Berta E, Lengyel I, Halmi S, Zrínyi M, Erdei A, Harangi M, et al. Hypertension in Thyroid Disorders. Front Endocrinol (Lausanne). 2019 Jul 17;10.

97. Levothyroxine, Euthyrox®. Summary of Product Characteristics. CBG Geneesmiddeleninformatiebank; 17-02-2023. Available from: https://www.geneesmiddeleninformatiebank.nl/smpc/h09009_smpc.pdf .

98. Levothyroxine sodium. Prescribing Information. Food and Drug Administration. 20/07/2023. Available from: https://nctr-crs.fda.gov/fdalabel/services/spl/set-ids/4f40db1e-3dd9-4c7b-93e6-64f0fd10d551/spl-doc?hl=levothyroxine .

99. He W, Li S, Zhang J an, Zhang J, Mu K, Li X ming. Effect of Levothyroxine on Blood Pressure in Patients With Subclinical Hypothyroidism: A Systematic Review and Meta-Analysis. Front Endocrinol (Lausanne). 2018 Aug 14;9.

100. Berwaerts J, Webster J. Analysis of risk factors involved in oral-anticoagulant-related intracranial haemorrhages. QJM. 2000 Aug 1;93(8):513–21.

101. Rivaroxaban. Xarelto®. Product Information. European Medicines Agency (EN); 23-02-2023. Available from: https://www.ema.europa.eu/en/documents/product-information/xarelto-epar-product-information_en.pdf .

102. Methylergometrine, Methergin®. Summary of Product Characteristics. CBG Geneesmiddeleninformatiebank; 19/07/2022. Available from: https://www.geneesmiddeleninformatiebank.nl/smpc/h03686_smpc.pdf .

103. Methylergonovine maleate. Prescribing Information. Food and Drug Administration. 17/02/2023. Available from: https://nctr-crs.fda.gov/fdalabel/services/spl/set-ids/70106231-37f5-3e64-e053-2991aa0ab9af/spl-doc?hl=methylergonovine .

104. Summary of Product Characteristics Methergin® (Methylergometrine). CBG Geneesmiddeleninformatiebank; 19:07:2022.

105. Xylometazoline, Otrivin®. Summary of Product Characteristics. CBG Geneesmiddeleninformatiebank; 17-02-2023. Available from: https://www.geneesmiddeleninformatiebank.nl/smpc/h21065_smpc.pdf .

106. Oxymetazoline. Prescribing Information. Food and Drug Administration. 11/07/2023. Available from: https://nctr-crs.fda.gov/fdalabel/services/spl/set-ids/2ee2b332-9b85-45a6-b242-3f0ebfbd08a6/spl-doc?hl=oxymetazoline .

107. Bromocriptine, Parlodel®. Summary of Product Characteristics. CBG Geneesmiddeleninformatiebank; 17-05-2022. Available from: https://www.geneesmiddeleninformatiebank.nl/smpc/h08202_smpc.pdf .

108. Bromoctiptine Mesylate, Parlodel®. Prescribing Information. Food and Drug Administration. 28/02/2023. Available from: https://nctr-crs.fda.gov/fdalabel/services/spl/set-ids/fc2a08dd-4fb6-4ac4-9082-f99552fae25c/spl-doc?hl=bromocriptine .

109. Kirsch C, Iffy L, Zito GE, McArdle JJ. The role of hypertension in bromocriptine-related puerperal intracranial hemorrhage. Neuroradiology. 2001 Apr 2;43(4):302–4.

110. Watson DL, Bhatia RK, Norman GS, Brindley BA, Sokol RJ. Bromocriptine mesylate for lactation suppression: a risk for postpartum hypertension? Obstetrics and gynecology. 1989 Oct;74(4):573–6.

111. Herings RMC, Stricker BHC. Bromocriptine and suppression of postpartum lactation. Pharmacy World & Science. 1995 Jul;17(4):133–7.

112. Gulleroglu K, Olgac A, Bayrakci U, Erdogan O, Kinik ST, Baskin E. Hyperprolactinemia as a Rare Cause of Hypertension in Chronic Renal Failure. Ren Fail. 2012 Jul 2;34(6):792–4.

113. Bernard N, Jantzem H, Becker M, Pecriaux C, Bénard-Laribière A, Montastruc J, et al. Severe adverse effects of bromocriptine in lactation inhibition: a pharmacovigilance survey. BJOG. 2015 Aug;122(9):1244–51.

114. Beck RA, Mercado DL, Seguin SM, Andrade WP, Cushner HM. Cardiovascular effects of pseudoephedrine in medically controlled hypertensive patients. Arch Intern Med. 1992 Jun;152(6):1242–5.

115. Adrenaline, Epipen®. Summary of Product Characteristics. CBG Geneesmiddeleninformatiebank; 10-11-2021. Available from: https://www.geneesmiddeleninformatiebank.nl/smpc/h32727_smpc.pdf .

116. Efedrine. Summary of Product Characteristics. CBG Geneesmiddeleninformatiebank; 18/01/2023. Available from: https://www.geneesmiddeleninformatiebank.nl/smpc/h123237_smpc.pdf .

117. Dopamine. Summary of Product Characteristics. CBG Geneesmiddeleninformatiebank; 05-12-2017. Available from: https://www.geneesmiddeleninformatiebank.nl/smpc/h116931_smpc.pdf.

118. Noradrenaline, Mykronor®. Summary of Product Characteristics. CBG Geneesmiddeleninformatiebank; 25/07/2022. Available from: https://www.geneesmiddeleninformatiebank.nl/smpc/h126921_smpc.pdf .

119. Epinephrine, Epipen®. Prescribing Information. Food and Drug Administration. 13/04/2023. Available from: https://nctr-crs.fda.gov/fdalabel/services/spl/set-ids/6ee4fecf-83b1-46f9-9b40-f8594710d2f5/spl-doc?hl=epinephrine .

120. Dopamine hydrochloride. Prescribing Information. Food and Drug Administration. 30/05/2023. Available from: https://nctr-crs.fda.gov/fdalabel/services/spl/set-ids/b61fffd0-7bc8-09e2-e053-2995a90ae0e4/spl-doc?hl=dopamine .

121. Ephedrine sulfate. Prescribing Information. Food and Drug Administration; 03/05/2023. Available from: https://nctr-crs.fda.gov/fdalabel/services/spl/set-ids/d89dd8d5-7698-61db-e053-2a95a90a764b/spl-doc?hl=ephedrine%20sulfate .

122. Laccourreye O, Werner A, Giroud JP, Couloigner V, Bonfils P, Bondon-Guitton E. Benefits, limits and danger of ephedrine and pseudoephedrine as nasal decongestants. Eur Ann Otorhinolaryngol Head Neck Dis. 2015 Feb;132(1):31–4.

123. Hadzic A, Vloka J, Patel N, Birnbach D. Hypertensive crisis after a successful placement of an epidural anesthetic in a hypertensive parturient. Case report. Reg Anesth. 1995;20(2):156–8.

124. Formoterol, Atimos®. Summary of Product Characteristics CBG Geneesmiddeleninformatiebank; 07/10/2022. Available from: https://www.geneesmiddeleninformatiebank.nl/smpc/h31994_smpc.pdf.

125. Isoprenaline. Summary of Product Characteristics. CBG Geneesmiddeleninformatiebank; 20/06/2022. Available from: https://www.geneesmiddeleninformatiebank.nl/smpc/h127536_smpc.pdf .

126. Dobutamine. Summary of Product Characteristics. CBG Geneesmiddeleninformatiebank; 18/10/2022. Available from: https://www.geneesmiddeleninformatiebank.nl/smpc/h19143_smpc.pdf .

127. Salbutamol, Ventolin®. Summary of Product Characteristics CBG Geneesmiddeleninformatiebank; 10/09/2020. Available from: https://www.geneesmiddeleninformatiebank.nl/smpc/h07686_smpc.pdf.

128. Formoterol fumarate. Prescribing Information. Food and Drug Administration; 24/03/2023. Available from: https://nctr-crs.fda.gov/fdalabel/services/spl/set-ids/35538d89-a984-4335-acdf-85a893f51eee/spl-doc?hl=formoterol%20fumarate .

129. Dobutamine. Prescribing Information. Food and Drug Administration; 19/05/2023. Available from: https://nctr-crs.fda.gov/fdalabel/services/spl/set-ids/74ba9408-17d3-48ac-be0b-a4fee9e7a1a5/spl-doc?hl=dobutamine .

130. Tullo V, Bussone G, Omboni S, Barbanti P, Cortelli P, Curone M, et al. Efficacy of frovatriptan and other triptans in the treatment of acute migraine of hypertensive and normotensive subjects: a review of randomized studies. Neurological Sciences. 2013 May 22;34(S1):87–91.

131. Sumatriptan, Imigran®. Summary of Product Characteristics). CBG Geneesmiddeleninformatiebank; 01/10/2021. Available from: https://www.geneesmiddeleninformatiebank.nl/smpc/h29414_smpc.pdf .

132. Sumatriptan Succinate. Prescribing Information. Food and Drug Administration. 07/06/2023. Available from: https://nctr-crs.fda.gov/fdalabel/services/spl/set-ids/d3b61201-fb84-446b-83a6-b72e8e1d6d04/spl-doc?hl=sumatriptan%20succinate .

133. Fleishaker JC, McEnroe JD, Azie NE, Francom SF, Carel BJ. Cardiovascular effect of almotriptan in treated hypertensive patients. Clin Pharmacol Ther. 2002 Mar;71(3):169–75.

134. Jhee S, Salazar D, Ford N, Fulmor I, Sramek J, Cutler N. A Double-Blind, Randomized, Crossover Assessment of Blood Pressure Following Administration of Avitriptan, Sumatriptan, Or Placebo To Patients with Mild To Moderate Hypertension. Cephalalgia. 1999 Mar 7;19(2):95–9.

135. Smith DA, Cleary EW, Watkins S, Huffman CS, Dilzer SC, Lasseter KC. Pharmacokinetics and Pharmacodynamics of Zolmitriptan in Patients with Mild to Moderate Hypertension: A Double-Blind, Placebo-Controlled Study. The Journal of Clinical Pharmacology. 1998 Aug;38(8):685–93.

136. Di Pauli F, Riedl K, Hegen H, Auer M, Berek K, Krajnc N, et al. Alemtuzumab induced hemodynamic change in relapsing multiple sclerosis occurs independent of corticosteroid premedication – a retrospective multicentre study. Mult Scler Relat Disord. 2022 Jul;63:103810.

137. Alemtuzumab, Lemtrada®. Product Information. European Medicines Agency (EN); 10/07/2023. Available from: https://www.ema.europa.eu/en/documents/product-information/lemtrada-epar-product-information_en.pdf .

138. Alemtuzumab, Lemtrada®. Prescribing Information. Food and Drug Administration. 23/05/2023. Available from: https://nctr-crs.fda.gov/fdalabel/services/spl/set-ids/6236b0bc-82e9-4447-9a78-f57d94770269/spl-doc?hl=lemtrada .

139. Shosha E, Casserly C, Tomkinson C, Morrow SA. Blood pressure changes during alemtuzumab infusion for multiple sclerosis patients. Eur J Neurol. 2021 Apr 5;28(4):1396–400.

140. Lenalidomide, Revlimid®. Product Information. European Medicines Agency (EN); 02-03-2023. Available from: https://www.ema.europa.eu/en/medicines/human/EPAR/revlimid#product-information-section .

141. Lenalidomide, Revlimid®. Prescribing Information. Food and Drug Administration. 24/03/2023. Available from: https://nctr-crs.fda.gov/fdalabel/services/spl/set-ids/5fa97bf5-28a2-48f1-8955-f56012d296be/spl-doc?hl=revlimid .

142. Basiliximab, Simulect®. Product Information. European Medicines Agency (EN); 27/02/2023. Available from: https://www.ema.europa.eu/en/documents/product-information/simulect-epar-product-information_en.pdf .

143. Basiliximab, Simulect®. Prescribing Information. Food and Drug Administration. 14/06/2023. Available from: https://nctr-crs.fda.gov/fdalabel/services/spl/set-ids/1af01887-b69d-444b-91ed-ebfe12784440/spl-doc?hl=simulect .

144. Zhang GQ, Zhang CS, Sun N, Lv W, Chen BM, Zhang JL. Basiliximab application on liver recipients: a meta-analysis of randomized controlled trials. Hepatobiliary & Pancreatic Diseases International. 2017 Apr;16(2):139–46.

145. Mancia G, Kreutz R, Brunström M, Burnier M, Grassi G, Januszewicz A, et al. 2023 ESH Guidelines for the management of arterial hypertension The Task Force for the management of arterial hypertension of the European Society of Hypertension. J Hypertens. 2023 Dec;41(12):1874–2071.

146. Bortezomib, Velcade®. Product Information Velcade® (Bortezomib). European Medicines Agency (EN); 04-06-2021. Available from: https://www.ema.europa.eu/en/documents/product-information/velcade-epar-product-information_en.pdf .

147. Bortezomib, Velcade®. Prescribing Information. Food and Drug Administration. 25/10/2022. Available from: https://nctr-crs.fda.gov/fdalabel/services/spl/set-ids/1521d321-e724-4ffc-adad-34bf4f44fac7/spl-doc?hl=velcade .

148. Hošková L, Málek I, Kopkan L, Kautzner J. Pathophysiological mechanisms of calcineurin inhibitor-induced nephrotoxicity and arterial hypertension. Vol. 66, Physiological Research. 2017. p. 167–80.

149. Cyclosporine, Neoral®. Summary of Product Characteristics CBG Geneesmiddeleninformatiebank; 24/05/2023. Available from: https://www.geneesmiddeleninformatiebank.nl/smpc/h17496_smpc.pdf.

150. Cyclosporine, Neoral®. Prescribing Information. Food and Drug Administration. 11/07/2023. Available from: https://nctr-crs.fda.gov/fdalabel/services/spl/set-ids/94461af3-11f1-4670-95d4-2965b9538ae3/spl-doc?hl=cyclosporin .

151. Marienhagen K, Lehner F, Klempnauer J, Hecker H, Borlak J. Treatment of cyclosporine induced hypertension: Results from a long-term observational study using different antihypertensive medications. Vascul Pharmacol. 2019 Apr;115:69–83.

152. Klein IHHT, Abrahams A, van Ede T, Hen?? RJ, Koomans HA, Ligtenberg G. Different effects of tacrolimus and cyclosporine on renal hemodynamics and blood pressure in healthy subjects. Transplantation. 2002 Mar;73(5):732–6.

153. Snanoudj R, Kriaa F, Arzouk N, Beaudreuil S, Hiesse C, Durrbach A, et al. Single-Center experience with cyclosporine therapy for kidney transplantation: analysis of a Twenty-Year period in 1200 patients. Transplant Proc. 2004 Mar;36(2):S83–8.

154. Higgins R. Hyponatraemia and hyperkalaemia are more frequent in renal transplant recipients treated with tacrolimus than with cyclosporin. Further evidence for differences between cyclosporin and tacrolimus nephrotoxicities. Nephrology Dialysis Transplantation. 2004 Feb 1;19(2):444–50.

155. Taylor DO, Barr ML, Radovancevic B, Renlund DG, Mentzer Jr RM, Smart FW, et al. A randomized, multicenter comparison of tacrolimus and cyclosporine immunosuppressive regimens in cardiac transplantation: decreased hyperlipidemia and hypertension with tacrolimus. The Journal of Heart and Lung Transplantation. 1999 Apr;18(4):336–45.

156. Woo M, Przepiorka D, Ippoliti C, Warkentin D, Khouri I, Fritsche H, et al. Toxicities of tacrolimus and cyclosporin A after allogeneic blood stem cell transplantation. Bone Marrow Transplant. 1997 Dec 1;20(12):1095–8.

157. Suttorp MM, Hoekstra T, Mittelman M, Ott I, Franssen CFM, Dekker FW. Effect of Erythropoiesis-Stimulating Agents on Blood Pressure in Pre-Dialysis Patients. PLoS One. 2013 Dec 31;8(12):e84848.

158. Epoetine alfa, Eprex®. Summary of Product Characteristics CBG Geneesmiddeleninformatiebank; 24/06/2021. Available from: https://www.geneesmiddeleninformatiebank.nl/smpc/h18479_smpc.pdf .

159. Epoetin-alfa, Epogen®. Prescribing Information - Food and Drug Administration. 25/07/2018. Available from: https://nctr-crs.fda.gov/fdalabel/services/spl/set-ids/1f2d0b28-9cc5-4523-80b8-637fdaf3f7a5/spl-doc?hl=Epogen .

160. Palmer SC, Saglimbene V, Mavridis D, Salanti G, Craig JC, Tonelli M, et al. Erythropoiesis-stimulating agents for anaemia in adults with chronic kidney disease: a network meta-analysis. Cochrane Database of Systematic Reviews. 2014 Dec 8;

161. Palmer SC, Navaneethan SD, Craig JC, Johnson DW, Tonelli M, Garg AX, et al. Meta-analysis: erythropoiesis-stimulating agents in patients with chronic kidney disease. Ann Intern Med. 2010 Jul 6;153(1):23–33.

162. Tonia T, Mettler A, Robert N, Schwarzer G, Seidenfeld J, Weingart O, et al. Erythropoietin or darbepoetin for patients with cancer. Cochrane Database of Systematic Reviews. 2012 Dec 12;

163. Tonelli M, Hemmelgarn B, Reiman T, Manns B, Reaume MN, Lloyd A, et al. Benefits and harms of erythropoiesis-stimulating agents for anemia related to cancer: a meta-analysis. Can Med Assoc J. 2009 May 26;180(11):E62–71.

164. Cody JD, Hodson EM. Recombinant human erythropoietin versus placebo or no treatment for the anaemia of chronic kidney disease in people not requiring dialysis. Cochrane Database of Systematic Reviews. 2016 Jan 20;

165. Abatacept, Orencia®. Product Information. European Medicines Agency (EN); 27/06/2023. Available from: https://www.ema.europa.eu/en/documents/product-information/orencia-epar-product-information_en.pdf .

166. Abatacept, Orencia®. Prescribing Information. Food and Drug Administration. 15/12/2021. Available from: https://nctr-crs.fda.gov/fdalabel/services/spl/set-ids/0836c6ac-ee37-5640-2fed-a3185a0b16eb/spl-doc?hl=orencia .

167. Leflunomide, Arava®. Product Information. European Medicines Agency (EN); 28-03-2022. Available from: https://www.ema.europa.eu/en/documents/product-information/arava-epar-product-information_en.pdf .

168. Teriflunomide, Aubagio®. Product Information. European Medicines Agency (EN); 14/07/2023. Available from: https://www.ema.europa.eu/en/documents/product-information/aubagio-epar-product-information_en.pdf .

169. Leflunomide, Arava®. Prescribing Information. Food and Drug Administration. 04/10/2022. Available from: https://nctr-crs.fda.gov/fdalabel/services/spl/set-ids/320f63f2-fac3-4aee-aff8-85724e00ef52/spl-doc?hl=leflunomide .

170. Teriflunomide, Aubagio®. Prescribing Information. Food and Drug Administration. 30/01/2023. Available from: https://nctr-crs.fda.gov/fdalabel/services/spl/set-ids/4650d12c-b9c8-4525-b07f-a2d773eca155/spl-doc?hl=aubagio .

171. Scott DL, Smolen JS, Kalden JR, van de Putte LB, Larsen A, Kvien TK, et al. Treatment of active rheumatoid arthritis with leflunomide: two year follow up of a double blind, placebo controlled trial versus sulfasalazine. Ann Rheum Dis. 2001 Oct;60(10):913–23.

172. Rozman B, Praprotnik S, Logar D, Tomsic M, Hojnik M, Kos-Golja M, et al. Leflunomide and hypertension. Ann Rheum Dis. 2002 Jun;61(6):567–9.

173. Ishaq M, Razzaque S, Shohail F, Kumar A, Muhammad JS. Onset of Hypertension in Leflunamide Treated Low Socioeconomic Rheumatoid Arthritis Patients: An Unseen Iceberg. Curr Rheumatol Rev. 2019 Jul 31;15(3):242–5.

174. Nurmohamed MT, van Halm VP, Dijkmans BAC. Cardiovascular Risk Profile of Antirheumatic Agents in Patients with Osteoarthritis and Rheumatoid Arthritis. Drugs. 2002;62(11):1599–609.

175. Tranylcypromine, Tracydal®. Summary of Product Characteristics. CBG Geneesmiddeleninformatiebank; 27-01-2021. Available from: https://www.geneesmiddeleninformatiebank.nl/smpc/h115752_smpc.pdf.

176. Tranylcypromine. Prescribing Information. Food and Drug Administration. 03/03/2023. Available from: https://nctr-crs.fda.gov/fdalabel/services/spl/set-ids/df892a65-52e4-8eb2-e053-2a95a90aa25f/spl-doc?hl=tranylcypromine .

177. Lavin MR, Mendelowitz A, Kronig MH. Spontaneous hypertensive reactions with monoamine oxidase inhibitors. Biol Psychiatry. 1993 Aug;34(3):146–51.

178. Taylor BP, Quitkin FM, McGrath PJ, Stewart JW. Do Antihypertensives Make Tranylcypromine Safer? J Clin Psychiatry. 2005 May 15;66(05):657–8.

179. Keck PE, Pope HG, Nierenberg AA. Autoinduction of hypertensive reactions by tranylcypromine? J Clin Psychopharmacol. 1989 Feb;9(1):48–51.

180. Zandee WT, Alsma J, Birkenhäger TK, den Meiracker AH VAN, van Hoek M, Versmissen J. [Hypertension en orthostatic hypotension during use of monoamine oxidase (MAO) inhibitors]. Tijdschr Psychiatr. 2017;59(6):366–71.

181. Bonnet U. Moclobemide: Therapeutic Use and Clinical Studies. CNS Drug Rev. 2003 Mar;9(1):97–140.

182. Yamada M, Yasuhara H. Clinical pharmacology of MAO inhibitors: safety and future. Neurotoxicology. 2004 Jan;25(1–2):215–21.

183. Sirolimus, Rapamune®. Product Information. European Medicines Agency (EN); 25-07-2022. Available from: https://www.ema.europa.eu/en/medicines/human/EPAR/rapamune#product-information-section .

184. Sirolimus, Rapamune®. Prescribing Information. Food and Drug Administration. 17/02/2023. Available from: https://nctr-crs.fda.gov/fdalabel/services/spl/set-ids/3275b824-3f82-4151-2ab2-0036a9ba0acc/spl-doc?hl=rapamune .

185. Liu YM, Shao YQ, He Q. Sirolimus for Treatment of Autosomal-Dominant Polycystic Kidney Disease: A Meta-Analysis of Randomized Controlled Trials. Transplant Proc. 2014 Jan;46(1):66–74.

186. Andreassen AK, Broch K, Eiskjær H, Karason K, Gude E, Mølbak D, et al. Blood Pressure in De Novo Heart Transplant Recipients Treated With Everolimus Compared With a Cyclosporine-based Regimen: Results From the Randomized SCHEDULE Trial. Transplantation. 2019 Apr;103(4):781–8.

187. Midodrine, Gutron®. Summary of Product Characteristics. CBG Geneesmiddeleninformatiebank; 02-07-2021. Available from: https://www.geneesmiddeleninformatiebank.nl/smpc/h16514_smpc.pdf .

188. Midodrine Hydrochloride. Prescribing Information. Food and Drug Administration. 14/04/2023. Available from: https://nctr-crs.fda.gov/fdalabel/services/spl/set-ids/60d5a132-9cfc-48e2-aa71-1949aeb255c3/spl-doc?hl=midodrine .

189. Stavert B, McGuinness MB, Harper CA, Guymer RH, Finger RP. Cardiovascular Adverse Effects of Phenylephrine Eyedrops. JAMA Ophthalmol. 2015 Jun 1;133(6):647.

190. Alpay A, Canturk Ugurbas S, Aydemir C. Efficiency and safety of phenylephrine and tropicamide used in premature retinopathy: a prospective observational study. BMC Pediatr. 2019 Dec 6;19(1):415.

191. Bhatia J, Varghese M, Bhatia A. Effect of 10% phenylephrine eye drops on systemic blood pressure in normotensive & hypertensive patient. Oman Med J. 2009 Jan;24(1):30–2.

192. Motta MMS, Coblentz J, Fernandes BF, Burnier Jr, MN. Mydriatic and Cardiovascular Effects of Phenylephrine 2.5% versus Phenylephrine 10%, Both Associated with Tropicamide 1%. Ophthalmic Res. 2009;42(2):87–9.

193. Parsaik AK, Singh B, Altayar O, Mascarenhas SS, Singh SK, Erwin PJ, et al. Midodrine for Orthostatic Hypotension: A Systematic Review and Meta-Analysis of Clinical Trials. J Gen Intern Med. 2013 Nov 18;28(11):1496–503.

194. Wecht JM, Weir JP, Katzelnick CG, Chiaravalloti ND, Kirshblum SC, Dyson-Hudson TA, et al. Double-blinded, placebo-controlled crossover trial to determine the effects of midodrine on blood pressure during cognitive testing in persons with SCI. Spinal Cord. 2020 Sep 17;58(9):959–69.

195. Bellew SD, Johnson KL, Nichols MD, Kummer T. Effect of Intranasal Vasoconstrictors on Blood Pressure: A Randomized, Double-Blind, Placebo-Controlled Trial. J Emerg Med. 2018 Oct;55(4):455–64.

196. Hatton RC, Winterstein AG, McKelvey RP, Shuster J, Hendeles L. Ambulatory Care: Efficacy and Safety of Oral Phenylephrine: Systematic Review and Meta-Analysis. Annals of Pharmacotherapy. 2007 Mar 29;41(3):381–90.

197. Gelotte CK, Zimmerman BA. Pharmacokinetics, Safety, and Cardiovascular Tolerability of Phenylephrine HCl 10, 20, and 30 mg After a Single Oral Administration in Healthy Volunteers. Clin Drug Investig. 2015 Sep 13;35(9):547–58.

198. Chrysant SG. Effectiveness and Safety of Phosphodiesterase 5 Inhibitors in Patients with Cardiovascular Disease and Hypertension. Curr Hypertens Rep. 2013 Oct 7;15(5):475–83.

199. Milrinone. Summary of Product Characteristics. CBG Geneesmiddeleninformatiebank; 24/09/2021. Available from: https://www.geneesmiddeleninformatiebank.nl/smpc/h119792_smpc.pdf .

200. Milrinone Lactate. Prescribing Information. Food and Drug Administration. 05/04/2023. Available from: https://nctr-crs.fda.gov/fdalabel/services/spl/set-ids/fde1e354-4f15-4ade-9ae3-db2ba67e0431/spl-doc?hl=milrinone .

201. Alhashemi JA, Hooper J. Treatment of milrinone — associated tachycardia with ß-blockers. Canadian Journal of Anaesthesia. 1998 Jan;45(1):67–70.

202. Chaudhri S, Kenny GNC. Nitroprusside-Sparing Effects of Enoximone. Cardiology. 1990;77(3):46–50.

203. Mincu RI, Mahabadi AA, Michel L, Mrotzek SM, Schadendorf D, Rassaf T, et al. Cardiovascular Adverse Events Associated With BRAF and MEK Inhibitors. JAMA Netw Open. 2019 Aug 9;2(8):e198890.

204. Binimetinib, Mektovi®. Product Information. European Medicines Agency (EN); 26/01/2022. Available from: https://www.ema.europa.eu/en/medicines/human/EPAR/mektovi#product-information-section .

205. Binimetinib, Mektovi®. Prescribing Information. Food and Drug Administration. 10/06/2022. Available from: https://nctr-crs.fda.gov/fdalabel/services/spl/set-ids/6c3408ac-d401-4925-8a03-26591afbc240/spl-doc?hl=mektovi .

206. Dummer R, Schadendorf D, Ascierto PA, Arance A, Dutriaux C, Di Giacomo AM, et al. Binimetinib versus dacarbazine in patients with advanced NRAS-mutant melanoma (NEMO): a multicentre, open-label, randomised, phase 3 trial. Lancet Oncol. 2017 Apr;18(4):435–45.

207. Dummer R, Ascierto PA, Gogas HJ, Arance A, Mandala M, Liszkay G, et al. Encorafenib plus binimetinib versus vemurafenib or encorafenib in patients with BRAF -mutant melanoma (COLUMBUS): a multicentre, open-label, randomised phase 3 trial. Lancet Oncol. 2018 May;19(5):603–15.

208. Gettinger SN, Bazhenova LA, Langer CJ, Salgia R, Gold KA, Rosell R, et al. Activity and safety of brigatinib in ALK-rearranged non-small-cell lung cancer and other malignancies: a single-arm, open-label, phase 1/2 trial. Lancet Oncol. 2016 Dec;17(12):1683–96.

209. Kim DW, Tiseo M, Ahn MJ, Reckamp KL, Hansen KH, Kim SW, et al. Brigatinib in Patients With Crizotinib-Refractory Anaplastic Lymphoma Kinase–Positive Non–Small-Cell Lung Cancer: A Randomized, Multicenter Phase II Trial. Journal of Clinical Oncology. 2017 Aug 1;35(22):2490–8.

210. Camidge DR, Kim HR, Ahn MJ, Yang JCH, Han JY, Lee JS, et al. Brigatinib versus Crizotinib in *ALK* -Positive Non–Small-Cell Lung Cancer. New England Journal of Medicine. 2018 Nov 22;379(21):2027–39.

211. Yoshida S, Takeuchi T, Kotani T, Yamamoto N, Hata K, Nagai K, et al. Infliximab, a TNF-α inhibitor, reduces 24-h ambulatory blood pressure in rheumatoid arthritis patients. J Hum Hypertens. 2014 Mar 5;28(3):165–9.

212. Adalimumab, Humira®. Product Information. European Medicines Agency (EN); 11/10/2022. Available from: https://www.ema.europa.eu/en/medicines/human/EPAR/humira#product-information-section .

213. Infliximab, Remicade®. Prescribing Information. Food and Drug Administration. 08/04/2022. Available from: https://nctr-crs.fda.gov/fdalabel/services/spl/set-ids/a0a046c1-056d-45a9-bfd9-13b47c24f257/spl-doc?hl=remicade .

214. Desai RJ, Solomon DH, Schneeweiss S, Danaei G, Liao KP, Kim SC. Tumor Necrosis Factor-α Inhibitor Use and the Risk of Incident Hypertension in Patients with Rheumatoid Arthritis. Epidemiology. 2016 May;27(3):414–22.

215. Ruiz Garcia V, Burls A, Cabello JB, Vela Casasempere P, Bort-Marti S, Bernal JA. Certolizumab pegol (CDP870) for rheumatoid arthritis in adults. Cochrane Database of Systematic Reviews. 2017 Sep 8;2017(9).

216. Grossman C, Bornstein G, Leibowitz A, Ben-Zvi I, Grossman E. Effect of tumor necrosis factor-α inhibitors on ambulatory 24-h blood pressure. Blood Press. 2017 Jan 2;26(1):24–9.

217. Merlo G, Cozzani E, Burlando M, Calvieri S, Potenza C, Stingeni L, et al. Effects of TNFα inhibitors in patients with psoriasis and metabolic syndrome: a preliminary study. Giornale Italiano di Dermatologia e Venereologia. 2020 Feb;155(1).

218. Bhargava P. VEGF kinase inhibitors: how do they cause hypertension? American Journal of Physiology-Regulatory, Integrative and Comparative Physiology. 2009 Jul;297(1):R1–5.

219. Moslehi JJ. Cardiovascular Toxic Effects of Targeted Cancer Therapies. New England Journal of Medicine. 2016 Oct 13;375(15):1457–67.

220. Aflibercept, Zaltrap®. Product Information. European Medicines Agency (EN); 21/12/2022. Available from: https://www.ema.europa.eu/en/documents/product-information/zaltrap-epar-product-information_en.pdf .

221. Aflibercept, Zaltrap®. Prescribing Information. Food and Drug Administration. 25/11/2020. Available from: https://nctr-crs.fda.gov/fdalabel/services/spl/set-ids/f6725df6-50ee-4b0a-b900-d02ba634395d/spl-doc?hl=zaltrap .

222. Liu B, Ding F, Liu Y, Xiong G, Lin T, He D, et al. Incidence and risk of hypertension associated with vascular endothelial growth factor receptor tyrosine kinase inhibitors in cancer patients: a comprehensive network meta-analysis of 72 randomized controlled trials involving 30013 patients. Oncotarget. 2016 Oct 11;7(41):67661–73.

2 Supplementary Data – Translated assessment report

Hypertension: corticosteroids

Abbreviations:
CI, confidence interval
DBP, diastolic blood pressure
DMARD, disease-modifying anti-rheumatic drug
HR, hazard ratio
IQR, interquartile range
IRR, incidence rate ratio
OR, odds ratio
RA, rheumatoid arthritis
SBP, systolic blood pressure

Date of literature search: 24-08-2020

CONCLUSIONS
Corticosteroids can cause hypertension or worsen existing hypertension. This is especially the case in doses higher than 7.5 mg prednisolone or equivalent doses or in drugs with a pronounced mineralocorticosteroid effect.
It is possible that the rise in blood pressure is less prominent in patients already taking antihypertensive drugs.

OTHER REMARKS
Mineralocorticosteroids act on the distal renal tubules and promote reabsorption of water and salt, thereby increasing blood pressure. Glucocorticosteroids have mineralocorticosteroid potency as well, but in varying degrees. Other blood pressure-increasing effects of corticosteroids include:
- Redistribution of body fluids with increased plasma volume and increased cardiac output
- Increased sensitivity of blood vessels to catecholamines
- Activation of the central and peripheral nervous system
- A direct effect on smooth muscle tissue of blood vessels

Cortisone, hydrocortisone and fludrocortisone have a pronounced mineralocorticosteroid effect in addition to glucocorticosteroid action. The other corticosteroids have only limited mineralocorticosteroid potential.

Clinical relevance
Change in blood pressure was the main outcome of interest during assessment. Increases in blood pressure of 10 mmHg SBP and 5 mmHg DBP were considered clinically relevant. In patients with risk factors, an increase of 5 mmHg SBP was considered clinically relevant.
Decrease in blood pressure was considered clinically relevant if this was more than 20 mmHg SBP or 10 mmHg DBP.

PICO

P(atient)	Patients with hypertension
I(ntervention)	Corticosteroid (systemic therapy)
C(omparison / Control)	Placebo (or patients without hypertension)

O(utcome)	Effect on hypertension

Search:
PUBMED: (‘hypertension’[MeSH] OR (blood pressure)) AND (beclomethasone OR betamethasone OR budesonide OR cortisone OR dexamethasone OR fludrocortisone OR hydrocortisone OR methylprednisolone OR prednisolone OR prednisone OR triamcinolone)

OTHER – not compliant with PICO
There were no studies that complied with PICO. The following studies described the effect of corticosteroids on blood pressure in normotensive patients.

Reference	Results/remarks
Distler A et al. Studies on the mechanism of mineralocorticoid-induced blood pressure increase in man. Clin Sci (Lond) 1979;57(5):303s-305s<br><br>**Prospective clinical study**	**Results**<br>• Normotensive healthy volunteers (arterial blood pressure: 79 mmHg) were administered 0.8 mg/day fludrocortisone for 6 weeks. Arterial blood pressure rose significantly with 6 mmHg after 1 week fludrocortisone and bodyweight increased with 2 kg. The peripheral resistance and heart rhythm decreased, but the central venal pressure remained the same. After 6 weeks of treatment, blood pressure increased with 18 mmHg, peripheral resistance was increased and heart rhythm was decreased. The increase in blood pressure was caused at first by increased stroke volume and later on by increased peripheral resistance.
Miyabe Y et al. Amelioration of the adverse effects of prednisolone by rituximab treatment in adults with steroid-dependent minimal-change nephrotic syndrome. Clin Exp Nephrol 2016;20:103-110<br><br>**Prospective cohort study**	**Results**<br>• Patients (n = 54, age 28.2±10.4 years) with corticosteroid-sensitive nephrotic syndrome were treated with rituximab at t = 0, 6, 12 and 18 months. During the treatment period, maintenance therapy with prednisolone was tapered from 24.7±14.1 mg/day at t = 0 to 0.7±2.2 mg/day at t = 24 months.<br>• Systolic blood pressure decreased from 120.9±14.1 mmHg at t = 0 to 111.8±13.8 mmHg (p = 0.03). Diastolic blood pressure decreased from 74.4±12.9 mmHg to 70.3±11.6 mmHg during the same period (p = 0.04). The number of patients on antihypertensive medications decreased from 15 to 5 (p = 0.03).<br><br>**Remarks authors**<br>• Lower exposure to prednisolone caused a decrease in side effects after 24 months.
Panoulas VF et al. Long-term exposure to medium-dose glucocorticoid therapy associates with hypertension in patients with rheumatoid arthritis.	**Results**<br>• Of 398 patients with rheumatoid arthritis, 70% of whom had hypertension, 281 used no or limited prednisolone, 58 used long-term low-dose prednisolone (< 7.5 mg/day, > 6 months) and 59 used long-term medium-dose prednisolone (7.5-30 mg/day, > 6 months). The latter group used an average of 9.17 mg prednisolone per day at the time of study.

Rheumatology 2008;47:72-75<br><br>**Prospective cohort study**	• The prevalence of hypertension was significantly higher in the medium-dose prednisolone group (84.7%) than in the no- or limited-dose prednisolone group (67.3%) and the low-dose prednisolone group (70.7%).<br>• The OR for hypertension was increased for the group with medium-dose prednisolone compared with the group with no or limited-dose prednisolone. The OR remained elevated after adjustment for hypertension risk factors (OR=2.57; 95% CI: 1.01-6.56) and adjustment for disease characteristics of rheumatoid arthritis (OR=3.64; 95% CI:1.36-9.77).
Baker JF et al. Initiation of disease-modifying therapies in rheumatoid arthritis is associated with changes in blood pressure. J Clin Rheumatol 2018;24:203-209<br><br>**Retrospective cohort study**	**Results**<br>• Database study on the influence of DMARDs on blood pressure in veterans with rheumatoid arthritis (RA). Prednisone (n = 10,027, 76% with hypertension) was one of the studied DMARDs. Blood pressure was determined 6 months before treatment initiation, just after and 6 months after.<br>• The percentage of patients with blood pressure <130/90 mmHg was greater 6 months after starting the DMARD than at baseline (50.0% vs. 47.3%, p <0.001). A similar trend was seen for prednisone (p < 0.0001).<br>• There was a limited decrease in systolic and diastolic blood pressure 6 months after start of 1.67 and 0.60 mmHg, respectively (p < 0.05). In the subgroup with patients not taking antihypertensive drugs, there was only a significant decrease in systolic blood pressure, estimated at 0.75 mmHg (p < 0.05).<br>• A clinically relevant increase in blood pressure was seen more frequently with prednisone than with methotrexate (OR 1.18; 95%CI 1.10 to 1.27). A clinically relevant increase was described as >20 mmHg SBP or >10 mmHg DBP. The risk for hypertension was also greater with prednisone compared to methotrexate (HR 1.30; 95% CI 1.08 to 1.56).<br><br>**Remarks authors**<br>• Treatment with DMARDs leads to a decrease in blood pressure after starting treatment. This is also true for prednisone. In patients with RA, prednisone may have a beneficial effect on blood pressure because it inhibits RA-induced inflammation.<br><br>**Remarks assessors**<br>• Of the patients on prednisone, 73% were treated with an antihypertensive before starting prednisone. It is not known whether the dose of this antihypertensive was adjusted after starting prednisone.<br>• The dose of prednisone was not reported.
Bloechliger M et al. Adverse event profile of oral corticosteroids among	**Results**<br>• For each side effect of corticosteroids in asthmatics, a separate cohort and case-control study were performed in this database

asthma patients in the UK: cohort study with a nested case-control analysis. Respiratory Research 2018;19:75<br><br>**Retrospective cohort study**	study. One of the adverse events was the occurrence of hypertension, defined as a diagnosis code for hypertension and a prescription for an antihypertensive drug.<br>• In almost all cases, prednisolone was used as a corticosteroid. The incidence rate ratio (IRR) for hypertension was increased for prednisolone users (IRR 1.2; 95%CI 1.2 to 1.3) and former users (IRR 1.4; 95%BI 1.3 to 1.4) compared with patients who never used prednisolone. However, after adjusting for confounders, the case-control study found no association between prednisolone use and hypertension.<br><br>**Remarks authors**<br>• Other studies did find a significant association. This was not the case in this study, perhaps due to the definition of hypertension (both indication code and antihypertensive drug).
Rice JB et al. Quantitative characterization of the relationship between levels of extended corticosteroid use and related adverse events in a US population. Current Medical Research and Opinion 2018;34(8):1519-1527<br><br>**Retrospective cohort study**	**Results**<br>• Cohort study of occurrence of adverse events with corticosteroid use. Three cohorts were distinguished: intermittent use (<60 days) and continuous use (≥60 days) in low (≤7.5 mg/day), medium (>7.5 to ≤15 mg/day) or high (>15 mg/day) doses of prednisone equivalent.<br>• Hypertension was more common in the cohorts with continuous use (high dose 41.90% and IRR 2.42; medium dose 35.05% and IRR 2.02; low dose 33.91% and IRR 1.96; all $p < 0.001$) than with intermittent use (17.34%).<br><br>**Remarks assessor**<br>• Patients in the cohorts with continuous use were older than those with intermittent use. In addition, the prevalence of conditions differed in the cohorts, so confounding by indication cannot be ruled out. Also, no control group was included in this study.
Fardet L et al. Synthetic glucocorticoids and early variations of blood pressure: a population-based cohort study. J Clin Endocrinol Metab 2015;100(7):2777-2783<br><br>**Retrospective cohort study**	**Results**<br>• Cohort study of the influence of corticosteroids on blood pressure in the first 3 months after corticosteroid initiation.<br>• In patients treated with antihypertensive therapy before corticosteroid initiation, no increase in blood pressure was seen. In patients without antihypertensive therapy, a clinically irrelevant increase in SBP was seen (increase <1 mmHg, $p = 0.03$). No increase was seen for DBP ($p = 0.52$).<br>• Among patients without antihypertensive therapy, 10% had a rise in SBP >20 mmHg and 4% had a rise ≥30 mmHg. A similar number of patients had such a decrease in blood pressure.<br>• Patients with a large increase were older, thinner and had a history of hypertension. In addition, they were treated with prednisone/prednisolone more often.

	**Remarks authors**<br>• In the first three months of use, blood pressure increase with corticosteroids is not clinically relevant. The risk of extreme blood pressure rise is higher with prednisone/prednisolone, probably due to the high mineral corticoid potency.<br><br>**Remarks assessor**<br>• Patients on antihypertensive therapy were not studied further because little variation in blood pressure was seen.
Sazliyana S et al. Implications of immunosuppressive agents in cardiovascular risks and carotid intima media thickness among lupus nephritis patients. Lupus 2011;20:1260-1266<br><br>**Retrospective cohort study**	**Results**<br>• In this retrospective study in Malaysia, an association between hypertension and long-term corticosteroid use was seen in patients with lupus nephritis (n=82). In patients with hypertension (n= 55), the cumulative dose was 25.25 g (IQR 22.6 g) and in patients without hypertension 12.5 g (IQR 14.1 g) ($p < 0.001$). The duration of treatment with corticosteroids was 6.8 years (IQR 6.7 years) in patients with hypertension versus 3.25 years (IQR 3.9 years) in patients without hypertension ($p < 0.05$).<br>• Patients on pulse methylprednisolone treatment actually had lower systolic and diastolic blood pressure.
Buchman AL. Side effects of corticosteroid therapy. J Clin Gastroenterol 2001;33(4):289-294<br><br>**Review**	Systolic blood pressure increases by about 15 mmHg with a dose of 80-200 mg/day of cortisol (or equivalent). It may occur within 24 hours.<br>However, no increase in blood pressure occurs at 40 mg/day.
Sholter DE, Armstrong PW. Adverse effects of corticosteroids on the cardiovascular system. Can J Cardiol 2000;16:505-11<br><br>**Review**	Human studies are small and often lack a control group. 200 mg hydrocortisone for 1 week in 8 healthy volunteers gave an increase in blood pressure. 40 mg/day i.v. prednisolone along with methylprednisolone, triamcinolone and dexamethasone in equivalent doses was administered for 5 days in 6 healthy volunteers. Systolic blood pressure increased 6-13 mmHg and diastolic 7-11 mmHg. In a retrospective study of 195 rheumatoid arthritis patients taking prednisone (< 20 mg/day) for more than 1 year, no blood pressure change occurred. Blood pressure rise is mainly dependent on dosage, not on duration of therapy.
De Leeuw PW. Drug-induced hypertension – recognition and management in older patients. Drugs&Aging 1997;11(3):178-185<br><br>**Review**	Fludrocortisone may cause a sharp rise in blood pressure, especially in patients with pre-existing orthostatic hypertension and the elderly. Blood pressure rise has also been seen after intranasal use. The effect is reversible.

Guidelines

Reference	Results/remarks

Dutch guidelines [1-5]	Glucocorticosteroids could increase the risk for hypertension in several patient populations. At the start of treatment, regular control of blood pressure is indicated.

[1] Hakvoort L, Dubbeld P, Ballieux MJP, Dijkstra RH, Meijman HJ, Weisscher PJ, Willemse BG, Eizenga WH. NHG-Standaard Polymyalgia rheumatica en arteriitis temporalis (February 2010)
[2] Federatie Medisch Specialisten. Diagnostiek en behandeling van ernstig astma - Systemische corticosteroïden als langdurige behandeling van ernstig astma (version July 2020
[3] Federatie Medisch Specialisten. Diagnostiek en behandeling van COPD-longaanval in het ziekenhuis - Systemische corticosteroïden als behandeling bij COPD (April 2017)
[4] Federatie Medisch Specialisten. Jicht - Effectieve en veilige methoden om een acute jichtaanval te behandelen (February 2014)
[5] Federatie Medisch Specialisten. Hypertensie in de tweede en derde lijn - Diagnostiek secundaire hypertensie (December 2017)

SmPC

**Reference**	**Results/remarks**
SmPC Prednison Teva 31-10-2017‡	**4.4 Special warnings and precautions for use**<br>Because of the possibility of fluid retention, care must be taken when corticosteroids are administered to patients with renal insufficiency or hypertension or congestive heart failure.<br><br>Corticosteroids may worsen diabetes mellitus, osteoporosis, hypertension, glaucoma and epilepsy and therefore patients with these conditions or a family history of them should be monitored frequently.<br><br>**4.8 Undesirable effects**<br>Not known: hypertension
SPC Celestone 27-04-2018*	**4.4 Special warnings and precautions for use**<br>Particular care is required when considering the use of systemic corticosteroids in patients with the following conditions and frequent patient monitoring is necessary.<br>[…] Hypertension
SPC Fludrace 08-08-2017	**4.4 Special warnings and precautions for use**<br>Special caution must be observed in patients who seem to be predisposed to developing complications on the basis of hypertension<br><br>Particular care is required when considering the use of systemic corticosteroids in patients with the following conditions and frequent patient monitoring is necessary.<br>[…] Hypertension<br><br>**4.8 Undesirable effects**<br>Hypertension.

SPC Solu-Cortef 22-04-2020**	**4.4 Special warnings and precautions for use**<br>Adverse effects of glucocorticoids on the cardiovascular system, such as dyslipidaemia and hypertension, may predispose treated patients with existing cardiovascular risk factors to additional cardiovascular effects, if high doses and prolonged courses are used.<br><br>**4.8 Undesirable effects**<br>Hypertension

‡ Comparable information in SmPC Prednisolon Ratiopharm 03-10-2018, * Comparable information in SmPC Oradexon (14-05-2018), SmPC Kenacort-A40 28-05-2018** Comparable information in SmPC Solu-Medrol 17-07-2020

RISKFACTORS

	Uncontrolled, untreated hypertension

	Contra-indication	Action	Date
**Conclusion**	Yes	Yes	26-10-2020
